# Unveiling the impact of competition weight loss on gut microbiota: alterations in diversity, composition, and predicted metabolic functions

**DOI:** 10.1080/15502783.2025.2474561

**Published:** 2025-03-03

**Authors:** Anastasiia Driuchina, Ville Isola, Juha J Hulmi, Vera M Salmi, Jukka Hintikka, Juha P Ahtiainen, Satu Pekkala

**Affiliations:** a University of Jyväskylä, Faculty of Sport and Health Sciences, Jyväskylä, Finland; b Turku University Hospital, Department of Clinical Microbiology, Turku, Finland

**Keywords:** Fat loss, physique athletes, diet, sports nutrition, gut microbiome

## Abstract

**Background:**

Competitive sports and sports nutrition, popular among amateur athletes aiming for a lean physique, have limited research on gut microbiota.

**Methods:**

We conducted a 46-week study to analyze the consequences of fat loss and diet restrictions in 23 fitness athletes who prepared for a physique competition. Body composition, dietary intakes, serum cytokines and chemokines, and fecal samples were analyzed.

**Results:**

Fat loss through caloric restriction and aerobic exercise led to an increased phylogenetic diversity of gut microbiota and changes in the composition of gut microbiota, with *Faecalibacterium*, Lachnospiraceae, *Bacteroides*, and *Intestinimonas* showing altered abundances. Fat loss also changed the predicted microbial functions responsible for the metabolism of carbohydrates and amino acids. Consumption of energy, carbohydrates, fiber, vitamins and minerals, and various fatty acids decreased during the preparation for the competition, which was partly associated with changes in gut microbiota. Several cytokine levels decreased (IL1a, IL1b, IL10, and TFNα), and certain chemokine levels increased (GROa and RANTES). During the 23-week regain period after the competition, gut microbiota showed signs of recovery, with increased diversity compared to pre- and post-competition measurements. Most taxonomic changes returned to their baseline levels after the regain period.

**Conclusions:**

The study highlights the dynamic nature of gut microbiota and its response to fat loss and regain in non-obese fitness/physique competitors and provides novel insights into how competitive sports and sports nutrition can influence the gut ecosystem.

## 1. Introduction

Quick, strict dieting is rarely sustainable and can lead to a cycle of weight loss and subsequent regain, commonly called yo-yo dieting or weight cycling. This can occur when a person repeatedly loses weight and gains it back, usually through periods of strict dieting and normal or excessive eating. Weight cycling has long been considered unhealthy and may be more deleterious than steady weight gain alone [1,2]. It has been shown that short-term weight gain induces strong inflammatory and hypertrophic cardiomyopathy signatures in blood, and some of the changes can be reversed after weight loss, while most weight gain-induced changes persist [3]. A study in rodents shows that yo-yo eating leads to such changes in the gut microbiota (GM) that it contributes to accelerated post-dieting weight regain [4]. Studies have demonstrated the potentially negative impact of weight cycling; however, they have focused on obese individuals. Studies on weight cycling in normal-weight individuals are scarce, partly due to ethical questions about subjecting such individuals to a very low-calorie diet and a hard exercise regimen.

Beyond the typical yo-yo dieter, athletes are a group of people who experience weight-cycling due to intense episodic periods of training and caloric restriction before the competition [5–7]. Athletes may experience weight loss differently than individuals with obesity and sedentary individuals. Unlike the casual dieter, athletes are usually already/initially normal-weight and may be more likely to retain more muscle mass when losing weight [5–7] and often gain muscle (together with fat) during the off-season [1]. Weight loss through increased energy expenditure, decreased caloric intake, altered rates of metabolism, or other means can affect many different biological processes, including inflammation [8], cardiac function [9,10], and metabolism [11].

Many studies have investigated the effects of exercise on GM in overweight and obese individuals [12]. Studies have also shown that GM can influence weight loss in overweight individuals and predict individual weight loss and metabolic improvement upon dieting and exercise, respectively [13,14]. One study conducted by Hjorth et al. [15] showed that the GM composition of normal-weight individuals was associated with their ability to lose weight in response to dietary modifications. In addition, it has been shown that GM slightly responds to short-term weight gain and subsequent weight loss in overweight individuals [3].

Competition-oriented sports to obtain and maintain a lean and muscular physique have grown in popularity in recent years. This strategy is particularly popular among athletes who compete in aesthetic-focused or weight-bearing sports like bodybuilding, physique sports, athletics, wrestling, or boxing. These athletes frequently follow rigorous training and dietary regimens to gain muscle mass and strength and reduce body fat [5–7]. In addition, after the competition, they voluntarily regain their fat mass, leading to cyclical changes in body composition [6]. To our knowledge, the effects of physique sports on GM have not been extensively studied before. While the characteristics of the athletic GM have been reviewed by Mohr and coworkers [16], little is known about GM in conditions of undernutrition or low energy availability [17,18], especially in athletes. Thus, the novelty of this study lies in being one of the first to examine gut microbiota in aesthetic sports (such as physique/fitness/bodybuilding sports) following extreme diet and exercise regimens along with precise diet reporting. In this context, physique sports provide an intriguing lens through which we can examine the associations between GM, fat loss, and metabolic health in non-obese individuals. Investigations into the impact of competitive dieting and exercise on body composition and metabolic health have shown that while there are temporary effects from weight loss on metabolism, most changes are reversed after weight is regained [3,6,19–21]. A pilot study of five male bodybuilders showed individual responses to competition in circulating metabolites and GM [22]. Nevertheless, probably due to a small sample size and individual changes in GM, no significant effects of the competition preparation and post-competition in the composition or the diversity of GM were seen [22]. Thus, a comprehensive understanding of the underlying mechanisms, including GM changes, is still developing.

To fill in the knowledge gaps, we explored the effects of competition preparation fat loss on GM of physique athletes in a 46-week longitudinal study [19]. Various outcomes, such as body composition, serum cytokines and chemokines, diet, GM diversity and composition, and predicted GM functions, were measured at two time points: before and after preparation for the competition. Additionally, GM diversity was analyzed after the recovery from the competition.

## 2. Materials and methods

### 2.1. Study design and participants

This is a sub-study of a previously published work [19]. Briefly, the participants were 23 physique competitors who aimed to reduce fat mass and maintain muscle mass for physique competition. All competitors were required to compete in the Finnish Fitness Sports Association’s 2019 national championships and to be registered with the national doping control organization under WADA. The exclusion criteria were chronic diseases, using prescribed medications (except contraceptive pills) or any substances prohibited by WADA, competing in the junior (<19 years old) or master (>40 years old) categories or in a non-drug tested competition, having competed within 6 months before the first measurement or planning to compete within 6 months after the last measurement. All participants followed their preferred diet and exercise regimen. Baseline measurements were obtained before the 23-week dieting phase for the competition, then 1 week before the competition, and finally after the 23-week recovery (weight regain) period, during which the participants were advised to continue their regular training and diet regimen. Participants arrived at the laboratory between 07.00 and 09.00 am at the same time on each visit. The participants arrived for testing after an 8 h food and fluid fast. Participants were also instructed to sleep for 8 h, abstain from alcohol and caffeine for 12 h, and abstain from exercise for 24 h before the blood sampling.

The study was approved by the Ethics Committee of the Central Finland Health Care District (19 U/2018), and it complied with the guidelines of the Declaration of Helsinki. The participants were given a full explanation of the study design, protocols, and potential risks. All participants gave informed consent.

**Figure 1.**
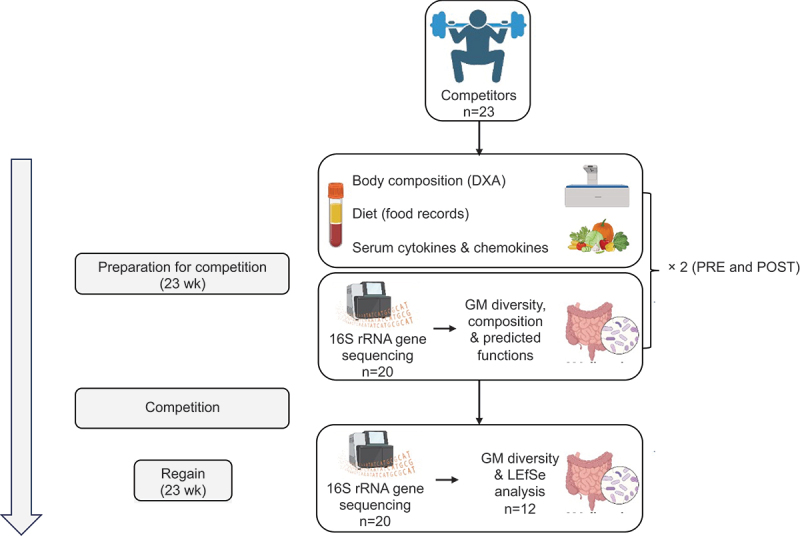
Study design of the sub-study, sample and data collection, and their analyses. DXA, dual-energy X-ray absorptiometry; GM, gut microbiota; LEfSe, linear discriminant analysis effect size. The figure was prepared using BioRender.

Figure 1 presents the study design and data analyses of this sub-study.

### 2.2. Measurement of body composition, diet, and inflammatory factors

The body composition of 23 participants was assessed with dual-energy X-ray absorptiometry (DXA, Prodigy; GE Lunar Corp., Madison, WI, USA) as described before [19].

Daily energy and macronutrient intake and dietary supplementation guidance were provided to the athletes by their coaches throughout the study. The competitors regularly provided nutrition logs, where they recorded the nutrition information about their diets. When athletes changed their nutrition, they reported these adjustments to the investigators. The diets were analyzed using the AivoDiet dietary analysis software (Flow Team Oy, Oulu, Finland). Nutrients in food supplements were included in the analysis.

The procedure for collecting blood samples has been described before [19]. The serum levels of cytokines (tumor necrosis factor-alpha (TNF-α), interleukin (IL)-1α, IL-1β, IL-6, IL-10 and interferon-gamma (IFN-γ)) and chemokines (Eotaxin, GROa, I-309, IL-8, IP-10, monocyte chemoattractant protein-1 (MCP-1), MCP-2, RANTES, TARC (CCL17) of 22 participants were analyzed before and after preparing to the competition. The cytokines were analyzed using a 6-plex Quansys ELISA assay (Human Cytokine Panel 1 #115249HU), and the chemokines using a 9-plex Quansys ELISA assay (Human Chemokine #120249HU) (Quansys Biosciences, Logan, UT, USA). Both assays were quantified with Quansys and Q-View software (Quansys Biosciences) as specified in the manufacturer’s instructions. The detection limits were as follows: TNF-α 1.2 pg/ml, IL-1α 0 pg/ml, IL-1β 1.95 pg/ml, IL-6 0.9 pg/ml, IL-10 0 pg/ml, IFN-γ 5.51 pg/ml, Eotaxin 0.04 pg/ml, GROa 0.92 pg/ml, I-309 0.47 pg/ml, IL-8 0.94 pg/ml, IP-10 0.54 pg/ml, MCP-1 0.28 pg/ml, MCP-2 0.95 pg/ml, RANTES 0.89 pg/ml, TARC 0.51 pg/ml and CCL17 0.65 pg/ml. IL-6 levels were under the detection limit in the analysis. Serum cytokine values were divided into quartiles, and a score from 1 (lowest quartile) to 4 (highest quartile) was assigned. The inflammatory score of each participant was the sum of each pro-inflammatory cytokine (TNF-α, IL-1α, IL-1β, IL-6) scores from which IFN-γ and IL-10 scores were subtracted.

### 2.3. Collection and analyses of the fecal samples

The competitors were asked to provide a fecal sample. After excluding samples from individuals who had an antibiotic course within 1 month prior to sampling, 20 samples (12 males and 8 females) before and after the preparation for the competition were included in the analyses. In addition, 12 samples (7 males and 5 females) were collected after weight regain. Due to known inter-individual variation in GM [23,24], intragroup comparisons are more informative than comparisons to a control group. The participants collected the fecal samples at their homes in OMNI fecal collection tubes (DNA Genotek, Ottawa, Canada). These tubes store stabilized DNA at ambient temperature for 60 days and thus allowed shipping the samples at room temperature. Upon arrival, the samples were immediately frozen and stored at −80°C until processing.

The total bacterial DNA was extracted, and the 16S rRNA gene was amplified and sequenced as described previously [25]. Briefly, an aliquot of the samples was homogenized accompanied by bead beating, and the total DNA was extracted using semi-automated GenoXtract (Nehren, Germany). For the 16S rRNA gene sequencing, the V3 and V4 regions of the 16S rRNA gene were amplified, and the sequencing was done with Ion Torrent PGM (Thermo Fisher, Waltham, MA, USA).

For the taxonomic analyses, the sequencing reads were processed using the DADA package for R (v4.0) [26]. First, fastQC and MultiQC were run to assess read quality. The reads were filtered and trimmed with the parameters trimLeft = 15, truncLen = 250, maxEE = 2, truncQ = 2. The error rates were estimated using the learnErrors-function, and error correction was run using the dada-function with the parameters HOMOPOLYMER_GAPPENALTY = −1, BAND_SIZE = 32. This was followed by clustering of amplicon sequence variants (ASVs) and removal of chimeras. Finally, a taxonomy was assigned to the amplicon sequence variants (ASVs) using the IDTAXA-function in the DECIPHER-package for R (v2.22) [27]. We predicted the Kyoto Encyclopedia of Genes and Genomes (KEGG) functions from the ASVs using MicFunPredict (available at: https://github.com/microDM/MicFunPred) and analyzed the data using Microbiomeanalyst (available at: https://www.microbiomeanalyst.ca).

### 2.4. Statistical analyses

The statistical analyses were performed using IBM SPSS Statistics 26 (Armonk, NY, USA), except for GM. Means and standard deviations (SD) were calculated for all test variables. The normal distribution of the variables was analyzed using Shapiro-Wilk’s test. Changes in the continuous variables were analyzed using paired t-test for normally distributed data and Wilcoxon signed-rank test for non-normally distributed data. We used an analysis of variance (ANOVA) for normally distributed and Mann-Whitney U test for non-normally distributed data to compare the differences between the sex groups. The statistical significance was set at *p* < .05.

The statistical analyses of GM were performed using R (v4.0) and the package mia (v1.9). Faith’s phylogenetic diversity was chosen as an alpha-diversity metric because it incorporates evolutionary relationships among taxa, providing a measure of phylogenetic richness within the community. Changes in GM alpha-diversity measures and phylogenetic diversity were analyzed with the Wilcoxon signed-rank test. Before the alpha-diversity analysis, the data was adjusted using rarefaction. The GM beta-diversity analysis was based on Bray-Curtis dissimilarity and PERMANOVA (Permutational Multivariate Analysis of Variance) for significance testing between the time points. The GM taxonomic changes between the pre- and post-competition measurements were analyzed with PERMANOVA. Differential abundance analysis was done using Wilcoxon signed-rank test after CLR-transformation. The statistical significance was set at FDR (false discovery rate) *p* < .05. Sex was included as a covariate in our statistical models to account for its potential influence on the observed associations between weight loss and microbiota changes. This adjustment ensured that the results primarily reflect the effects of the intervention while minimizing confounding by gender-related differences. In addition, we used LEfSe (Linear discriminant analysis Effect Size), as available in the galaxy module, to further explore taxonomic differences between the time points [28].

## 3. Results

### 3.1. The preparation to the competition decreased fat mass

This is a sub-cohort from the study of Isola et al. [19] consisting of the participants from which we were able to obtain fecal samples. Isola et al. [19] reported that energy intake decreased, and exercise volume increased which subsequently led to a decreased total fat mass. In our subgroup, we found that during the preparation for the competition lean/fat free mass remained unaltered, but fat mass decreased in all body parts (arms, legs, trunk, android, gynoid, total) (*p* < .001). Similarly, total mass and tissue mass decreased significantly in all body parts (arms *p* = .011, legs *p* = .001, rest *p* < .001). Sex did not influence the changes (Table 1).

**Table 1.** Body composition changes in the study participants. Pre-time point is before and post after preparing for the competition, *n* = 20 (12 males and 8 females).

		Pre	Post	*P*-value
Total body mass, kg	Male	94.5 ± 8.1	79.8 ± 6.2	<.001
	Female	69.0 ± 10.1	59.8 ± 7.9	<.001
	All	84.3 ± 15.5	71.8± 12.1	<.001
Total fat mass (arms, legs, trunk, android, gynoid), kg	Male	14.9 ± 5.1	4.7 ± 2.3	<.001
	Female	17.1 ± 6.0	6.6 ± 3.5	<.001
	All	15.9 ± 5.4	5.5 ± 2.9	<.001
Total fat, %	Male	16 ± 5	6 ± 3	<.001
	Female	24 ± 6	11 ± 5	<.001
	All	19 ± 7	8 ± 5	<.001

### 3.2. Dietary intakes of total energy and several macronutrients decreased during preparation to the competition

**Figure 2.**
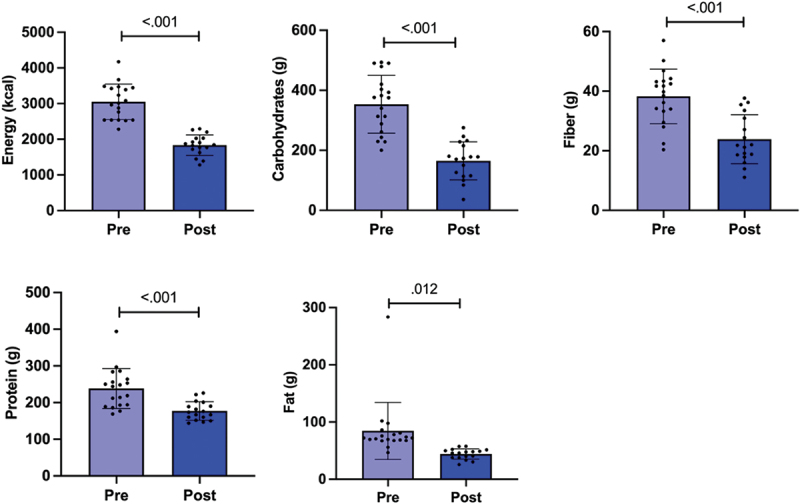
Dietary intakes of energy and macronutrients in the study participants. The bars show mean and 95% confidence interval (CI). The dots indicate individual values. Pre time point is before and post after preparing for the competition, *n*=20. *P*-value is indicated above the bars.

The intakes of total energy, carbohydrates, protein, fat, and fiber decreased during the preparation for the competition (*p* < .001 for total energy, carbohydrates, protein, and fiber, and *p* = .012 for fat, Figure 2). In addition, the consumption of saturated fatty acids (SFA) monounsaturated fatty acids (MUFA) and omega-6 series of polyunsaturated fatty acids (PUFA-N6) and linoleic acid decreased. When we looked at the sex differences, the consumption of total protein and fat decreased in males. In females, the percentage of energy derived from proteins increased and the consumption of trans-fatty acids decreased (Table S1).

The athletes consumed significantly less vitamins E and B1, phosphorus, magnesium, iron, zinc, and copper during the preparation for the competition compared to baseline (Table S2).

### 3.3. The serum levels of several cytokines decreased, and chemokines increased during preparation to the competition independent of fat mass changes

**Figure 3.**
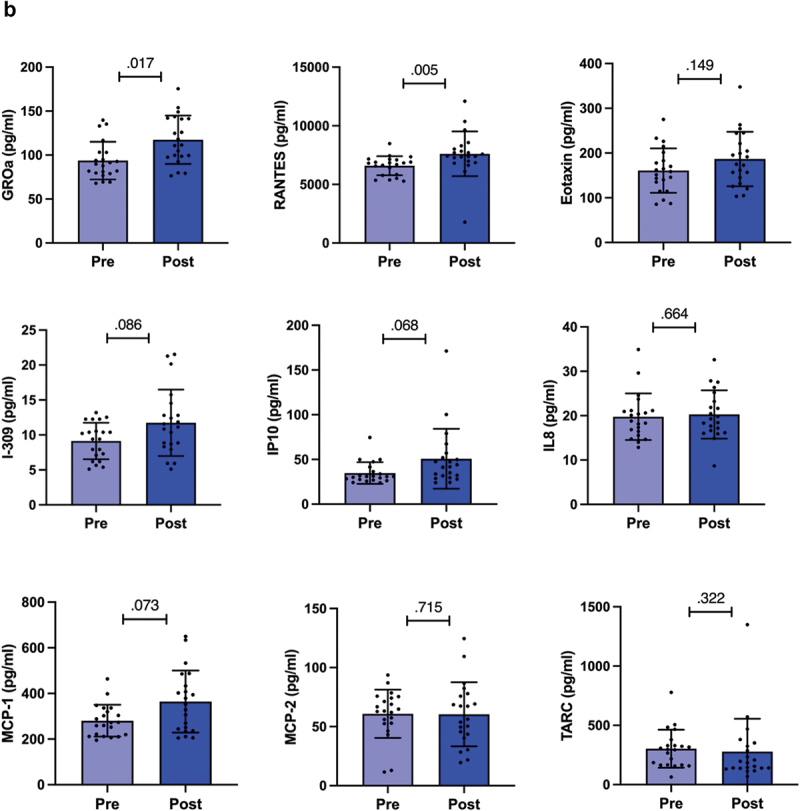
a) Serum levels of cytokines and inflammatory score. The inflammatory score is the sum of each pro-inflammatory cytokine (tnf-α, IL-1α, IL-1β, IL-6) scores from which ifn-γ and IL-10 scores were subtracted. b) Serum levels of chemokines. The bars show mean and 95% confidence interval (CI). The dots indicate individual values. Pre is before and post after preparing for the competition, *n* = 20. *P*-value is indicated above the bars.

Next, we analyzed the levels of serum cytokines and chemokines before and after competition weight loss. We found that the preparation to the competition was associated with a significant decrease in the cytokines IL1a, IL1b, IL10, and TFNα (*p* = .035, *p* = .005, *p* = .001, and *p* = .035 respectively, Figure 3a). No changes in the levels of IFN-γ were detected (Figure 3a) and the levels of IL6 were under the detection limit. Inflammatory score decreased during preparation to the competition (*p* = .036, Figure 3a). To understand whether the levels of the cytokines that are mainly secreted from the adipose tissue [29] were related to fat mass, we adjusted the results to total fat mass. We found that the differences between the time points remained the same suggesting that fat mass did not contribute to the changes in the cytokine levels.**Figure 3.**
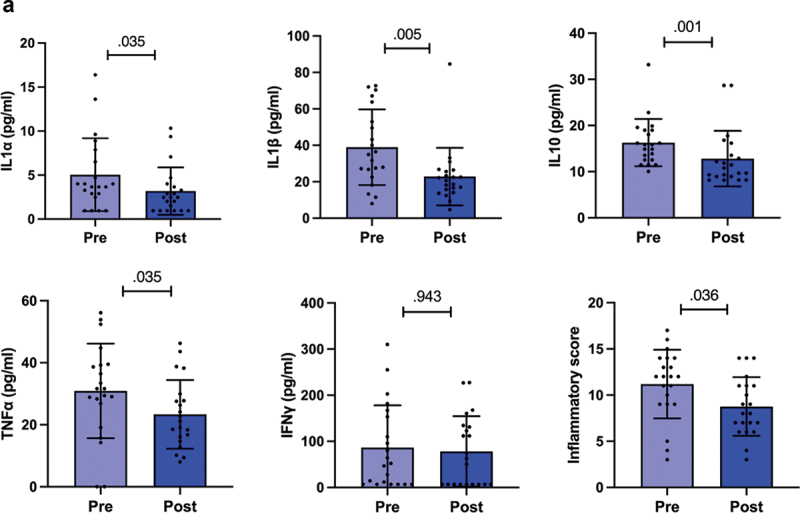

We found that there was an increase in the chemokines GROa and RANTES (*p* = .017 and *p* = .005 respectively, Figure 3b). After adjusting the results to total fat mass, the differences remained the same suggesting that fat mass did not contribute to the changes in the chemokine levels.

### 3.4. The phylogenetic diversity of the gut microbiota increased during fat loss

**Figure 4.**
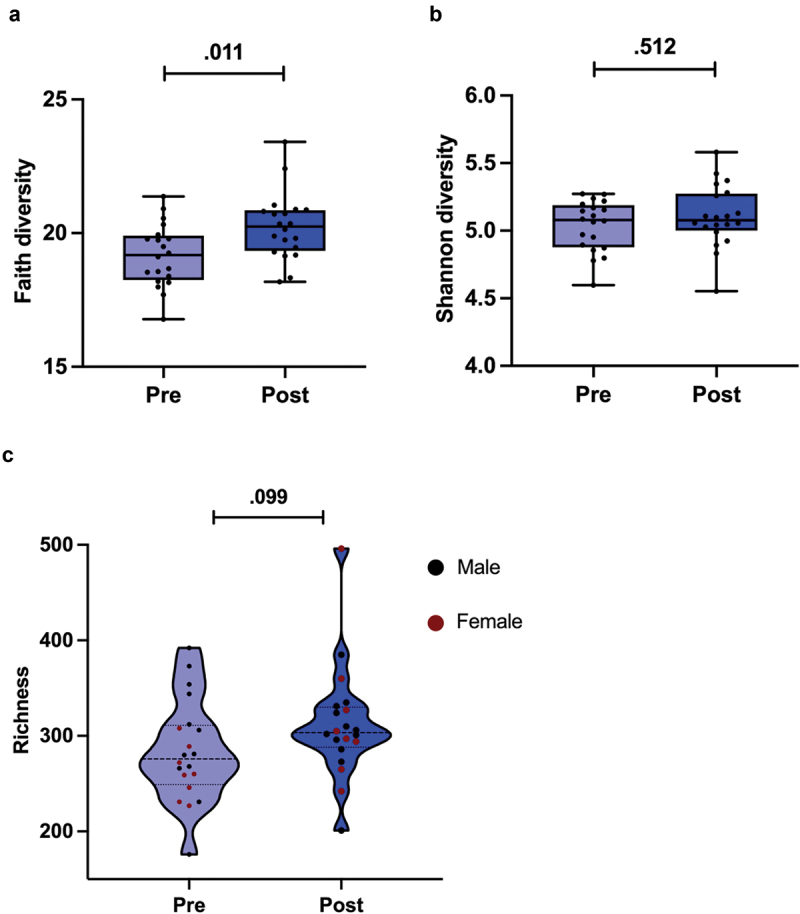
a) Faith index, which indicates phylogenetic diversity of GM. b) Shannon index, which indicates species diversity of GM. C) Chao1 index, which indicates GM species richness. Pre time point is before and post after competition preparation weight loss. The box plots show mean and 95% confidence interval (CI). The dots indicate individual values. *P*-value is indicated above the bars.

By analyzing the GM diversity, we found that the Faith index, which indicates phylogenetic diversity of GM, increased during the preparation for the competition (*p* = .011, Figure 4a). The difference in Shannon index (*i.e*., species diversity) or Chao1 (*i.e*., species richness) that indicate GM alpha-diversity between the time points was not significant (*p* = .512, Figure 4b, and *p* = .099, Figure 4c).

**Figure 5.**
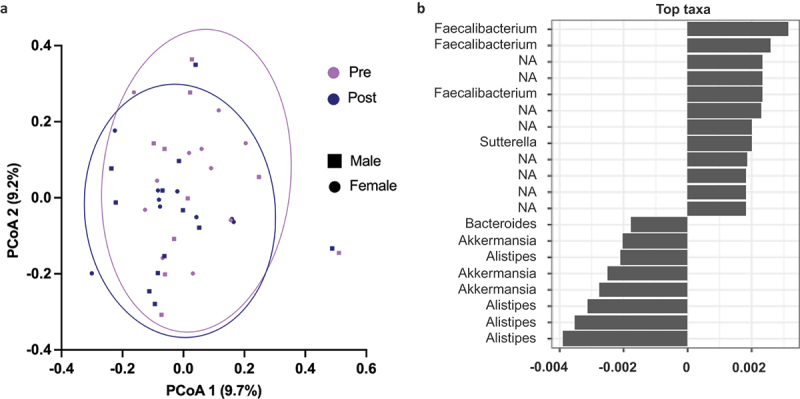
a) Bray-Curtis dissimilarity, which indicates beta-diversity of GM. Pre time point is before and post after competition preparation fat loss, *n*=20. The principal coordinate analysis (PCoA) showed a slight distinction between pre- and post-time points along the components. b) PERMANOVA coefficient plot showing the taxa whose abundances drive the differences between the time points. *p*=.0227. The initial plot showed taxa as code names, but for better readability we use genus level names. NA, non-identified taxa.

As shown by Bray-Curtis dissimilarity and PERMANOVA analyses, there were significant changes in GM beta-diversity (i.e. interindividual species diversity) during the preparation for the competition (Figure 5A, *p*=.042). The time variable was associated with the GM composition (*p*=.023). PERMANOVA coefficient plot visualizes the taxa whose abundances drive the differences between the time points, with the genera Alistipes and Faecalibacterium contributing most to the separation (Figure 5b).

### 3.5. Preparation for the competition changed the composition of the gut microbiota

**Figure 6.**
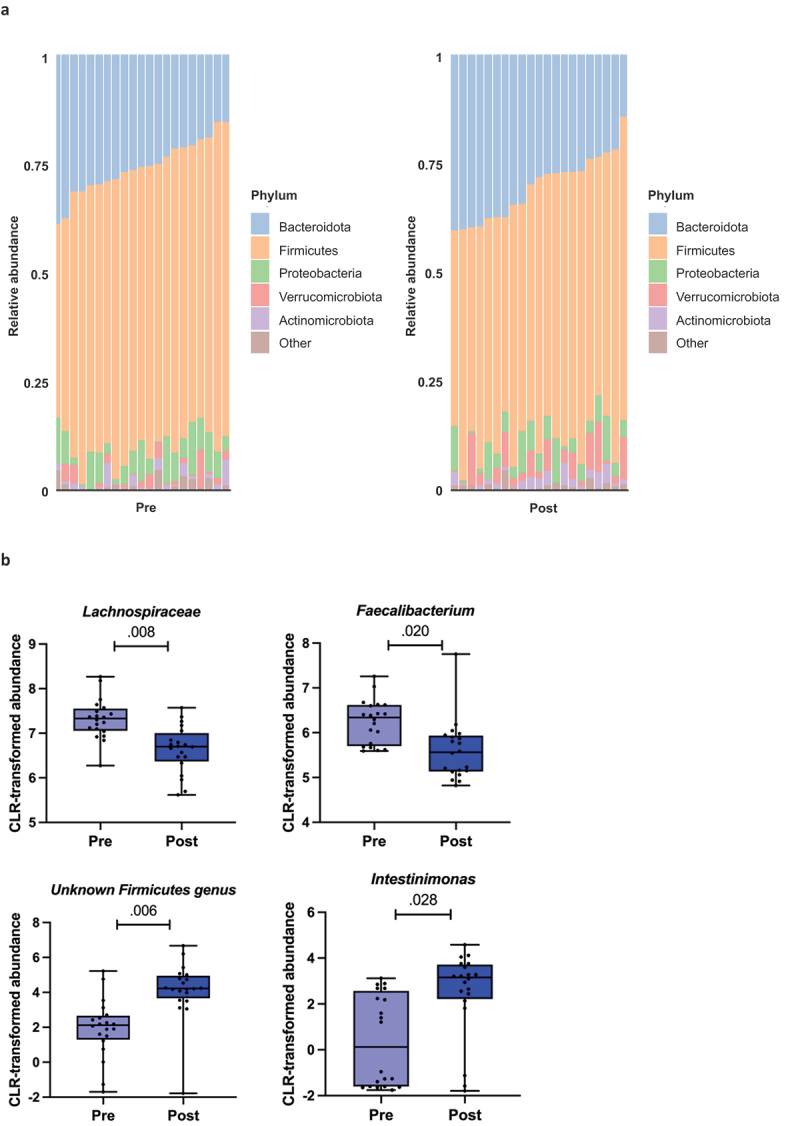
a) Phylum level composition of the gut microbiota before (pre) and after (post) preparing for the competition. b) the gut microbiota taxa that changed in response to the preparation for the competition. The bars show mean and 95% confidence interval (CI). The dots indicate individual values. Pre is before and post after preparing for the competition, *n*=20. *fdr*-value is indicated above the bars.

The GM composition of the individual samples at the phylum level before and after preparing for the competition is shown in Figure 6a. We further performed the differential abundance analysis to observe the changes in the GM composition during the preparation for the competition using Wilcoxon signed-rank test. The center log-transformed (CLR) abundances of the family Lachnospiraceae and the genus Faecalibacterium decreased (FDR=.008 and FDR = .020 respectively), while the abundances of the unknown Firmicutes genus and genus Intestinimonas increased (FDR=.006 and FDR = .028 respectively) (Figure 6b).

### 3.6. Numerous predicted microbial functions changed during the preparation to the competition

**Figure 7.**
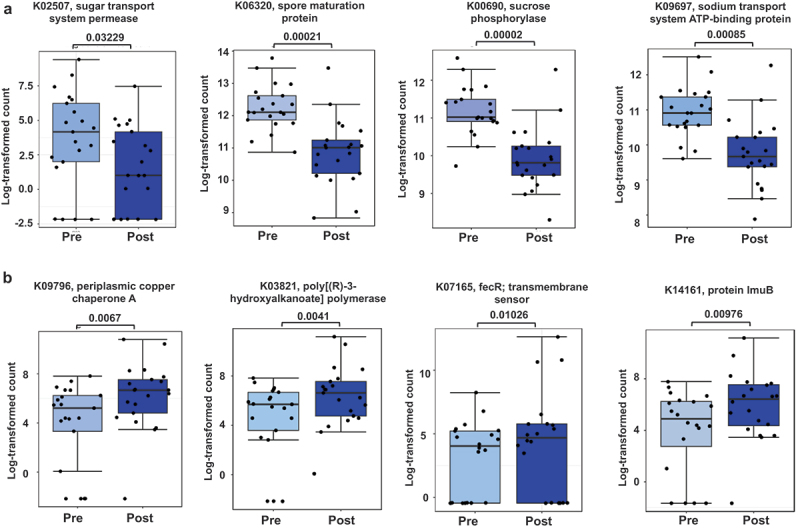
a) Most down-regulated and b) up-regulated predicted functions during weight loss. The box plots show mean and 95% confidence interval (CI). The dots indicate individual values. Pre is before and post after preparing for the competition. The predicted functional differences between the time points were analyzed with DeSeq2. *fdr*-value is indicated above the bars.

We predicted the Kyoto Encyclopedia of Genes and Genomes (KEGG) functions from the 16S rRNA gene sequences using MicFunPredict and analyzed the data using Microbiomeanalyst. After low count filtering (minimum count 4, and 20% prevalence in samples) and low variance filtering with interquartile range, a total of 4290 features remained. The DeSeq2 analysis revealed 988 significantly differing features between the time points (Table S3). The four most down-regulated predicted functions during the preparation to the competition are shown in Figure 7a, and the four most up-regulated predicted functions in Figure 7b. The pathway analysis shows that the predicted differential functions map into many metabolic pathways, including the metabolism of carbohydrates as well as degradation and biosynthesis of amino acids (Table 2).

**Table 2.** KEGG pathway analysis of the predicted differential functions altered during fat loss.

KEGG pathway	Hits	*P*-value
Oxidative phosphorylation	18	<.001
Starch and sucrose metabolism	13	<.001
Pantothenate and CoA biosynthesis	7	.002
Glyoxylate and dicarboxylate metabolism	11	.003
Porphyrin metabolism	11	.004
Geraniol degradation	3	.004
Citrate cycle (TCA cycle)	8	.004
Photosynthesis	8	.006
Leucine and isoleucine degradation	8	.006
Tyrosine and tryptophan biosynthesis	8	.008
Serine and threonine metabolism	11	.009
Butanoate metabolism	11	.009
Carbon fixation pathways in prokaryotes	10	.023
Aminobenzoate degradation	5	.023
Glycerophospholipid metabolism	5	.036
Tryptophan metabolism	5	.044

KEGG – Kyoto Encyclopedia of Genes and Genomes. Hits – the number of predicted differential functions that map into the given pathway.

### 3.7. Exercise energy expenditure, mean metabolic equivalent of task and diet may explain the GM compositional changes

**Figure 8.**
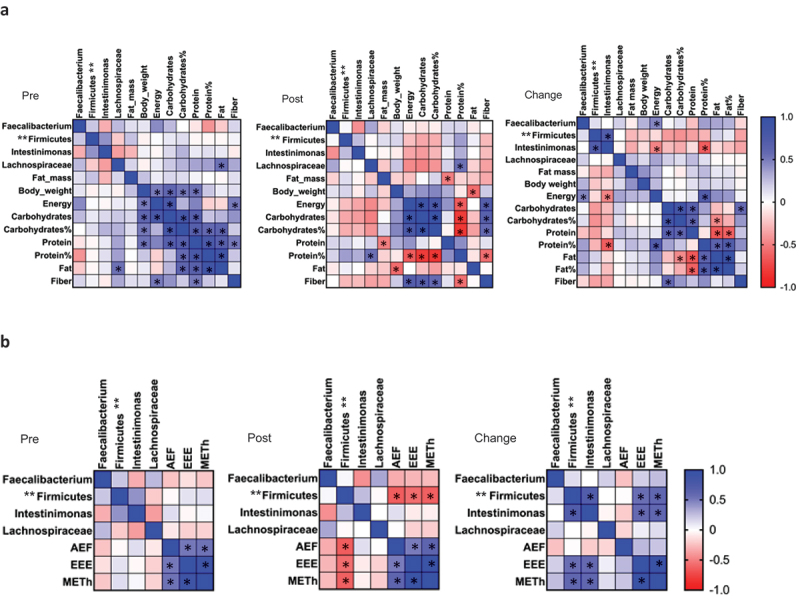
Associations between the CLR abundance of the four taxa and a) fat mass and dietary intakes, and b) exercise variables in response to the preparation for the competition. Pearson correlation was used for the analysis. AEF, aerobic exercise frequency, times per week. EEE, exercise energy expenditure, based on METh calculations. METh, mean metabolic equivalent of task, hours during the week. Pre is before and post after preparing for the competition, *n*=20. Change is the difference in values between pre and post. * *p* <.05, ** unknown Firmicutes genus.

We then examined the connections between the abundances of four specific microbial taxa, which abundance was affected by the competition weight loss (*Faecalibacterium*, unknown Firmicutes genus, *Intestinimonas*, and Lachnospiraceae) and several key variables. These included fat mass, body weight, dietary intake components (energy, carbohydrates, protein, fat, and fiber), cytokines and chemokines, and exercise metrics (aerobic exercise frequency, exercise energy expenditure, and mean metabolic equivalent of task – published before [19]) at two time points – before and after the competition preparation phase. In addition, we investigated the associations between changes in these abundances and variables (Figure 8).

Our analyses revealed that the body composition variables – specifically, total fat mass and body weight – did not exhibit significant associations with the microbial abundances, suggesting that the alterations occur independently of one another. However, shifts in energy intake were associated with changes in the abundances of *Faecalibacterium* and *Intestinimonas* (*p* = .044 and *p* = .032 respectively). Additionally, variations in relative protein intake were found to associate with *Intestinimonas* abundance changes (*p* = .003). Lachnospiraceae abundance demonstrated an association with fat intake before the competition (*p* = .020) and with relative protein intake after the competition (*p* = .030) (Figure 8a).

Further analysis revealed that the cytokine levels did not associate with the abundances of four of the microbial taxa (*Faecalibacterium*, unknown Firmicutes genus, *Intestinimonas*, and Lachnospiraceae) (Figure S1A). Only the changes in the levels of IFN-γ associated with the change in *Intestinimonas* abundance (*p* = .021). However, as we have pointed out before, the changes in the IFN-γ levels were insignificant.

On the other hand, changes in the levels of a few chemokines were associated with changes in microbial abundances. Specifically, shifts in GROa, RANTES, and MCP-2 levels were associated with changes in the abundances of *Faecalibacterium*, *Intestinimonas*, and Lachnospiraceae respectively (*p* = .047, *p* = .034, and *p* = .025) (Figure S1B). After the competition, MCP-2 levels demonstrated an association with *Faecalibacterium*, unknown Firmicutes genus, and *Intestinimonas* abundances (*p* = .032, *p* = .023, and *p* = .024 respectively), while GROa, IP-10, and I-309 levels associated with *Faecalibacterium*, unknown Firmicutes genus, and Lachnospiraceae (*p* = .036, *p* = .023, and *p* = .023 respectively) (Figure S1B).

Examining the exercise-related variables, our findings indicate that the changes in unknown Firmicutes genus and *Intestinimonas* abundances associated with exercise energy expenditure (*p* = .018 and *p* = .012 respectively) and mean metabolic equivalent of task (METh) (*p* = .011 and *p* = .010 respectively). Following the competition, unknown Firmicutes genus abundance alone exhibited associations with the aforementioned variables (*p* = .016 and *p* = .007) as well as with aerobic exercise frequency (*p* = .006) (Figure 8b). Therefore, these results suggest that the combined changes in diet and increase in exercise altered GM more than the changes in body composition.

### 3.8. The recovery of the gut microbiota diversity from the competition and enriched taxa

**Figure 9.**
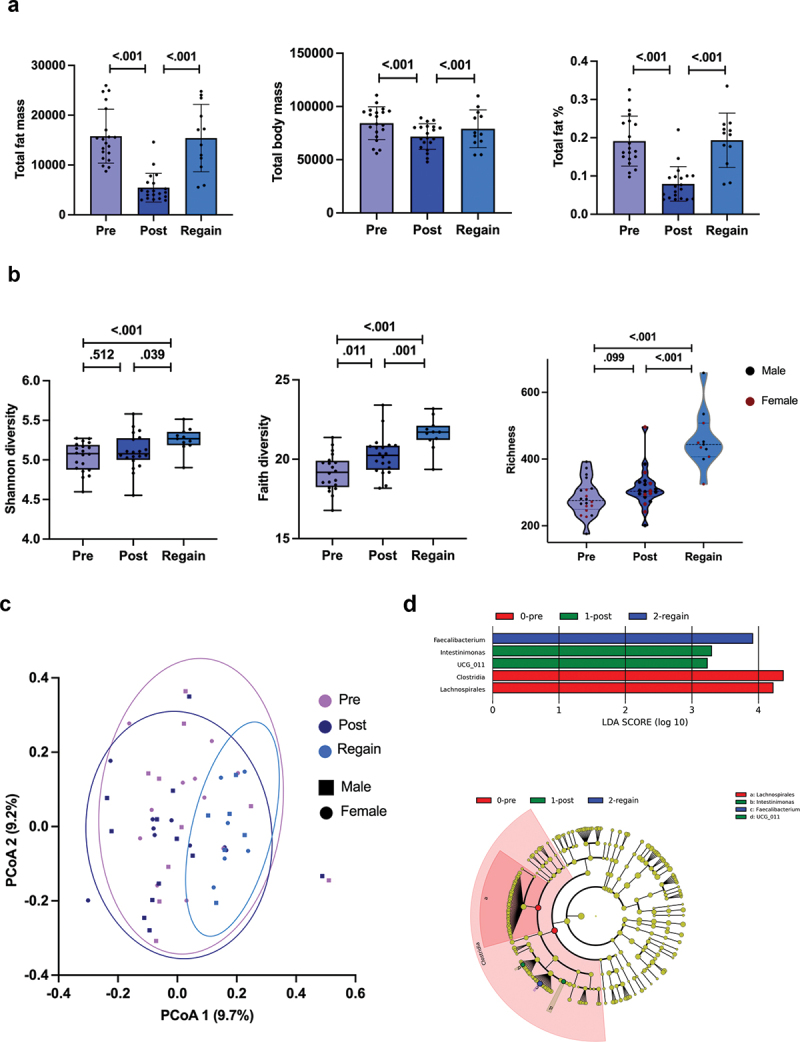
The recovery of the gut microbiota after competition. a) Body composition changes. b) Shannon, Faith, and Chao1 index. The box plots show mean and 95% confidence interval (CI). The dots indicate individual values. *P*-value is indicated above the bars. c) Principal coordinate analysis of the gut microbiota beta-diversity. The principal coordinate analysis (PCoA) showed a distinction between pre-, post-, and regain time points along the components. d) Enriched taxa from LEfSe analysis at different time points. LEfSe, linear discriminant analysis effect size. The LDA scores represent the effect size of each abundant species. The taxa enriched at each time point with an LDA score > 2 are considered significant. Pre time point is before and post after preparing for the competition, *n* = 20. The regain time point is 23 weeks after the competition, *n* = 12.

Because it is known that changes in diet can also very rapidly change GM [30], we were interested in knowing how GM recovers from the drastic diet changes and fat loss after a 23-week weight regain period following the competition. Collection and analysis of fecal samples was limited to 12 competitors due to the COVID-19 pandemic at the time of follow-up. The body composition variables returned to their baseline levels (Figure 9a). Interestingly, both Faith and Shannon indices increased compared to both pre (*p* < .001 for both, Figure 9b) and post-competition measurements (*p* = .014 and *p* = .03, respectively, Figure 9b). There was also a significant increase in Chao1 index after the regain period compared to pre- and post-competition measurements (*p* = .002 for both, Figure 9b). There was no difference between males and females in these GM diversity indices.

By analyzing Bray-Curtis dissimilarities, we found that GM beta-diversity (*i.e*., interindividual species diversity) increased significantly after weight regain (Figure 9c). No differences between males and females were seen (data not shown). Due to the uneven number of samples between time points, we used LEfSe analysis (Linear discriminant analysis Effect Size) to analyze taxonomic changes between the time points. We found that *Faecalibacterium* was enriched in recovery (*i.e*., regain) time point, while *Defluviitaleaceae UCG-011* and *Intestinimonas* were enriched during preparation to the competition. The LEfSe analysis further showed that Clostridia class and Lachnospirales order were enriched at baseline (Figure 9d). Therefore, weight regain was accompanied by significant changes in GM alpha-diversity, evidenced by increases in Faith, Shannon, and Chao1 indices, as well as an increase in beta-diversity and alterations in taxonomic abundance.

## 4. Discussion

In this study, we explored the physiological effects of physique competition preparation on athletes with a specific focus on how exercise and dietary energy restrictions were associated with changes in GM. We found that the preparation for the competition influenced not only the GM diversity and abundance of several dominant taxa but also changed several predicted microbial functions responsible for the metabolism of carbohydrates as well as amino acid biosynthesis and degradation. Following increased energy intake and subsequent weight regain, most of these shifts were restored.

Previous research has mainly concentrated on the impact of weight loss interventions on GM in overweight or obese populations, neglecting the potential implications for individuals within a normal weight range. One of the few studies in normal-weight individuals showed that the normal weight individuals with a higher abundance of *Prevotella* genus lost more body weight in response to dietary modifications than individuals with a lower abundance of *Prevotella* [15]. The present study addresses a critical research gap and contributes novel insights to the field by examining the effects of physique competition preparation on GM in non-obese individuals. Understanding how weight loss practices impact GM in athletic individuals can provide valuable information for health professionals, fitness enthusiasts, and individuals striving to achieve optimal health and body composition. We revealed an increase in the GM phylogenetic diversity, interindividual species diversity, and changes in the relative abundances of a few GM taxa – decrease of *Faecalibacterium* and Lachnospiraceae and increase in unknown Firmicute*s* genus and *Intestinimonas*. This is important as the GM composition and functions play a significant role in controlling various physiological functions, such as metabolism, immunological response, and general health [31].

Some past studies have shown how the combination of diet and exercise might affect the makeup of GM [32–34]. For example, a study by Clarke et al. [33] revealed that GM of rugby athletes had higher phylogenetic diversity than GM of same BMI-matched controls. Further, several gut microbial taxa were altered in the athletes, while the intakes of energy and nutrients were significantly higher in the athlete group [33]. A study conducted by Jang et al. [34] showed that an inadequately low-carbohydrate and low-fiber diet counteracts the benefits of exercise and high-protein diet on GM diversity. A review article by Beam et al. [32] examined the impact of various diets on the GM composition, revealing that plant-based and Mediterranean diets (low-fat and high-fiber) are the most beneficial to GM. Collectively, these studies suggest that there is a complex relationship between diet, exercise, and GM. Very little is known about calorie restriction in athletes [16,17]. However, athletes often restrict dietary fiber intake to prevent gastrointestinal disturbances [35]. One study examined how athlete dietary patterns (bodybuilders: high protein, high fat, low carbohydrate, and low dietary fiber diet; distance runners: low carbohydrate and low dietary fiber diet) associated with GM [34]. Contrary to our study, Jang et al. [34] found no effects of aerobic training associated with lower carbohydrate and fiber intake with the diversity of GM. However, in the bodybuilders the high fat intake was found to impact GM, while in our study no link between GM and fat intake was detected in physique athletes. The collective findings of these studies point to a complex interaction between nutrition, exercise, and GM. Future research should focus on identifying the specific dietary components and exercise routines that maximize GM health, especially in athletes, who may have distinct nutritional requirements.

We conducted detailed correlation analyses to gain insights into the relationships between various factors in the context of competition preparation. Specifically, we examined the associations between the abundances of four microbial taxa (*Faecalibacterium*, unknown Firmicutes genus, *Intestinimonas*, and Lachnospiraceae) that changed during the study and several key variables, including fat mass, body weight, dietary intakes, and exercise metrics at two time points, as well as the change in the taxonomic abundances and the variables between the time points. Our findings imply that specific microbial taxa may exhibit associations with certain aspects of dietary factors, such as energy and protein intakes. The changes in body composition did not exhibit significant associations with microbial abundances, suggesting that these alterations may occur independently of each other. Importantly, our analyses show that the abundances of unknown Firmicutes genus and *Intestinimonas* genus exhibited associations with exercise-related metrics, including exercise energy expenditure, mean metabolic equivalent of work, and aerobic exercise frequency. Altogether, these results suggest that the changes in diet quantity and quality as well as exercise altered GM more than changes in body composition. Our findings are supported by one study investigating the impact of lifelong strenuous physical activity on GM. The study revealed that GM of elderly athletes was characterized by a notably higher abundance of *Intestinimonas* compared to non-athlete elderly individuals [36]. It is known that several species of Lachnospiraceae, *Intestinimonas* and *Faecalibacterium* ferment carbohydrates and produce butyrate. Members of Lachnospiraceae family not only have a great capacity for carbohydrate metabolism, fatty acid synthesis, and degradation but also participate in branched-chain amino acid biosynthesis [37]. The phylum Firmicutes contains a broad spectrum of species with different metabolic capacities including production of bile salt and enhancing more fatty acids absorbed. We found using the LEfSe analysis between the time points that *Faecalibacterium* was enriched during the regain period and not during the preparation for the competition. While many studies have shown that exercise increases the abundance of this genus [12], our correlation analysis did not find a connection between the abundance of *Faecalibacterium* and exercise volume. Thus, more studies are needed to understand whether and why *Faecalibacterium* responds to the competition preparation in physique sports.

Although we did not analyze the fecal metabolome or metagenomes in this study, we found several predicted microbial functions that changed during the preparation for the competition. Further analysis of those functions revealed that most of them mapped into several metabolic pathways, including the metabolism of carbohydrates as well as the degradation and biosynthesis of amino acids. This could be related to decreased intakes of protein and carbohydrates in our study cohort, or in the changes in carbohydrate-metabolizing taxa.

A growing body of literature suggests that GM plays a significant role in modulating body fat and lean mass in the general population [38,39]. Our analyses, however, did not show significant associations between the changes in the abundances of four microbial taxa and the changes in body composition. This could be explained by the high degree of interindividual variability in GM and its sensitivity to a range of external factors, such as diet, exercise, and even genetic predispositions [40]. Despite the lack of associations in our study, the potential mechanisms by which GM could impact body composition remain of great interest.

Inflammatory and chemotactic signaling are essential for controlling the body’s immune response and tissue repair [41]. Strenuous exercise is known to increase the secretion of cytokines from the adipose tissue [42]. In the present study, we found a decline in several cytokine levels, which agrees with our previous studies showing a decrease in low-grade inflammation during preparation to physique competition [21,43]. These results may imply that additional factors, such as exercise intensity and nutrient intake, might have influenced the observed changes, as adjusting the analyses to fat mass did not affect the results. For instance, these changes might be explained by reduced energy, carbohydrates, and fiber consumed before the competition. In agreement, earlier studies have shown that low-carbohydrate and low-fiber diets have anti-inflammatory effects in different populations [33,44,45]. On the contrary, the weight loss of our athletes led to an increase in several chemokines. This finding is supported by a previous study showing increased chemokine levels in athletes following long-term exercise regimen [46]. However, the clinical significance of this finding and its relationship with athletes’ health and performance remain unknown. Further analysis revealed that there were no associations between the cytokine values and the following microbial taxa: unknown Firmicutes genus, *Intestinimonas*, Lachnospiraceae, and *Faecalibacterium*. However, we found that changes in these individual microbial taxa abundances were connected to fluctuations in certain chemokine levels, including GROa, RANTES, and MCP-2. While the variations in MCP-2 levels were not significant, GROa and RANTES levels showed associations with the abundances of the genera Faecalibacterium and *Intestinimonas*, respectively. The shift in pro-inflammatory IFNγ levels was also associated with the change in the abundance of *Intestinimonas*. *Intestinimonas* produces butyrate that may decrease inflammation and contribute to metabolic health. This finding suggests that certain cyto- and chemokines and the microbial abundances may have a complex connection in which they interact and possibly affect each other’s levels and activities within the body. Links between the shifts in the gut microbiota and cyto- and chemokines are a possible topic for future studies.

We set up this study to gain more insights into the effects of fat loss and dietary energy restrictions on GM of non-obese athletic individuals. The study highlights the intricate relationship between exercise, diet, and GM in the context of sports nutrition. The implications of our research for the field of GM are substantial. It emphasizes the importance of GM in adapting to the physiological demands of athletes, suggesting that rigorous training and dietary energy restrictions could induce reversible changes in GM. Ultimately, our research expands the boundaries of the gut microbiome studies into the field of sports science and sports nutrition. Identifying the relationships between the GM diversity and abundance as well as athlete-specific dietary and exercise regimens could lead to the possibility of creating personalized nutrition and training programs that maximize the benefits of the microbiome. Such personalized programs could have the potential not only to enhance athletes’ performance but also promote long-term health in athletes, establishing a novel approach to sports nutrition that is in harmony with the gut microbiome.

## Supplementary Material

Supplemental Material

## Data Availability

Access to the data is restricted due to personal information protection (General Data Protection Regulation (GDPR) 2016/679 and Directive 95/46/EC). However, it is possible to contact the corresponding author to request a copy of the material.
