# Supplementary material for: Unveiling the impact of competition weight loss on gut microbiota: alterations in diversity, composition, and predicted metabolic functions
Source: J Int Soc Sports Nutr. 2025 Mar 3;22(1):2474561. doi: 10.1080/15502783.2025.2474561 (PMC11881659; doi:10.1080/15502783.2025.2474561)
Supplement: Supplemental Material [file RSSN_A_2474561_SM5736.docx]

# **Supplementary materials**

**Table S1. Dietary intakes of energy and nutrients in competitors who had their GM analyzed (8 males and 8 females).** Pre time point is before and Post after preparing for the competition. SFA, saturated fatty acids. MUFA, monounsaturated fatty acids. PUFA, polyunsaturated fatty acids.

|  |  | Pre | Post | *P*-value |
| --- | --- | --- | --- | --- |
| Energy, kcal | Male | 3160±387 | 1899±286 | < .001 |
|  | Female | 2723±380 | 1779±318 | < .001 |
|  | Total | 2942±433 | 1839±299 |  |
| Carbohydrates, g | Male | 400±68 | 179±52 | < .001 |
|  | Female | 285±94 | 148±77 | < .001 |
|  | Total | 342±99 | 164±66 |  |
| Carbohydrates, % | Male | 51.6±8.9 | 37.7±6.5 | .002 |
|  | Female | 41.8±7.0 | 32.3±13.1 | .022 |
|  | Total | 46.7±9.3 | 35.0±10.4 |  |
| Fat, g | Male | 95.8±76.2 | 41.5±7.1 | .012 |
|  | Female | 74.3±15.0 | 47.5±10.5 | .178 |
|  | Total | 85.1±54.2 | 44.5±9.2 |  |
| Fat, % | Male | 28.8±26.0 | 19.9±2.8 | .187 |
|  | Female | 25.5±7.0 | 25.2±8.0 | .967 |
|  | Total | 27.1±18.5 | 22.6±6.4 |  |
| Protein, g | Male | 257±65 | 184±26 | .001 |
|  | Female | 204±28 | 172±25 | .091 |
|  | Total | 231±56 | 178±26 |  |
| Protein, % | Male | 33.5±10.5 | 39.9±7.4 | .112 |
|  | Female | 30.9±2.9 | 40.1±7.0 | .027 |
|  | Total | 32.2±7.5 | 40.0±7.0 |  |
| Saccharose, g | Male | 19.2±10.4 | 12.7±6.7 | .074 |
|  | Female | 17.2±10.5 | 14.8±11.7 | .491 |
|  | Total | 18.2±10.1 | 13.7±9.2 |  |
| Fiber, g | Male | 36.2±7.9 | 24.1±8.7 | .002 |
|  | Female | 38.3±10.8 | 24.0±8.8 | < .001 |
|  | Total | 37.2±9.2 | 24.0±8.5 |  |
| SFA, g | Male | 12.0±2.4 | 7.87±1.81 | .002 |
|  | Female | 14.3±4.7 | 7.58±2.67 | < .001 |
|  | Total | 13.1±3.8 | 7.72±2.21 |  |
| MUFA, g | Male | 22.0±5.1 | 13.5±3.0 | .005 |
|  | Female | 27.6±12.7 | 16.3±8.3 | < .001 |
|  | Total | 24.8±9.8 | 14.9±6.2 |  |
| PUFA, g | Male | 12.7±3.4 | 7.31±3.81 | .019 |
|  | Female | 12.1±4.8 | 9.96±5.74 | .319 |
|  | Total | 12.4±4.1 | 8.64±4.90 |  |
| FA20:5n-3 (EPA), mg | Male | 10.52±8.90 | 7.15±7.54 | .986 |
|  | Female | 133±281 | 395±1007 | .182 |
|  | Total | 72±202 | 201±717 |  |
| FA22:6n-3 (DHA), mg | Male | 224±77 | 159±70 | .530 |
|  | Female | 308±464 | 431±666 | .244 |
|  | Total | 266±324 | 295±479 |  |
| PUFA N3, g | Male | 2.82±0.91 | 1.94±0.66 | .261 |
|  | Female | 4.10±2.48 | 4.11±3.72 | .995 |
|  | Total | 3.46±1.92 | 3.03±2.82 |  |
| PUFA N6, g | Male | 10.60±2.84 | 5.98±3.18 | < .001 |
|  | Female | 9.35±3.11 | 6.13±2.03 | .004 |
|  | Total | 9.98±2.95 | 6.06±2.58 |  |
| Trans-FA, g | Male | 0.80±0.24 | 0.75±0.27 | .675 |
|  | Female | 0.85±0.24 | 0.58±0.22 | .032 |
|  | Total | 0.82±0.23 | 0.67±0.25 |  |
| Linoleic Acid, mg | Male | 10024±2902 | 5528±3185 | < .001 |
|  | Female | 8196±3250 | 5400±2029 | .012 |
|  | Total | 9110±3123 | 5464±2581 |  |
| A-Lipoic Acid, mg | Male | 1469±1048 | 886±714 | .076 |
|  | Female | 1910±2302 | 1571±1833 | .283 |
|  | Total | 1690±1743 | 1228±1389 |  |

**Table S2. Dietary intakes of vitamins and minerals in competitors who had their GM analyzed (8 males and 8 females).** Pre time point is before and Post after preparing for the competition.

|  |  | Pre | Post | *P*-value |
| --- | --- | --- | --- | --- |
| Vitamin A, µg | Male | 1206±585 | 1103±723 | .681 |
|  | Female | 1130±1161 | 1002±728 | .611 |
|  | Total | 1168±889 | 1052±703 |  |
| Vitamin D, µg | Male | 4.42±1.90 | 2.66±1.15 | .047 |
|  | Female | 3.49±2.59 | 2.34±1.59 | .176 |
|  | Total | 3.95±2.25 | 2.50±1.35 |  |
| Vitamin E, mg | Male | 16.1±5.1 | 9.9±2.1 | < .001 |
|  | Female | 13.5±3.0 | 10.5±2.5 | .027 |
|  | Total | 14.8±4.3 | 10.2±2.3 |  |
| Vitamin B1, mg | Male | 2.77±0.43 | 1.88±0.35 | < .001 |
|  | Female | 1.96±0.53 | 1.52±0.32 | .036 |
|  | Total | 2.37±0.62 | 1.70±0.37 |  |
| Vitamin B2, mg | Male | 2.95±0.89 | 2.45±0.60 | .037 |
|  | Female | 2.27±0.52 | 2.29±0.32 | .929 |
|  | Total | 2.61±0.79 | 2.37±0.47 |  |
| Niacin equivalents (NE), mg | Male | 88.7±18.1 | 78.5±12.4 | .085 |
|  | Female | 58.7±9.3 | 59.6±10.2 | .878 |
|  | Total | 73.7±20.8 | 69.0±14.7 |  |
| Pyridoxine, mg | Male | 4.05±0.93 | 3.51±0.53 | .085 |
|  | Female | 2.90±0.61 | 2.94±0.45 | .880 |
|  | Total | 3.47±0.96 | 3.23±0.56 |  |
| Folate, µg | Male | 504±150 | 458±223 | .517 |
|  | Female | 415±132 | 402±85 | .857 |
|  | Total | 460±144 | 430±165 |  |
| Vitamin B12, µg | Male | 9.18±2.02 | 7.67±1.20 | .035 |
|  | Female | 6.51±1.31 | 5.86±1.68 | .332 |
|  | Total | 7.84±2.15 | 6.76±1.69 |  |
| C vitamin, mg | Male | 182±102 | 245±170 | .316 |
|  | Female | 230±76 | 249±83 | .760 |
|  | Total | 206±90 | 247±129 |  |
| Calcium, mg | Male | 1304±2182 | 461±251 | .136 |
|  | Female | 869±349 | 544±248 | .551 |
|  | Total | 1086±1526 | 502±245 |  |
| Phosphorus, mg | Male | 2668±386 | 2017±412 | < .001 |
|  | Female | 2353±355 | 1750±312 | < .001 |
|  | Total | 2511±394 | 1884±379 |  |
| Potassium, mg | Male | 5492±1080 | 4948±1194 | .284 |
|  | Female | 4683±1198 | 4498±598 | .710 |
|  | Total | 5087±1178 | 4723±942 |  |
| Magnesium, mg | Male | 697±95 | 485±100 | .002 |
|  | Female | 544±115 | 419±114 | .044 |
|  | Total | 620±129 | 452±109 |  |
| Iron, mg | Male | 2824±474 | 1772±405 | < .001 |
|  | Female | 2091±443 | 1355±305 | < .001 |
|  | Total | 2457±583 | 1564±407 |  |
| Zinc, mg | Male | 2490±378 | 1818±450 | .002 |
|  | Female | 1850±362 | 1365±297 | .018 |
|  | Total | 2170±487 | 1591±436 |  |
| Copper, mg | Male | 269±32 | 176±42 | < .001 |
|  | Female | 219±46 | 146±45 | .002 |
|  | Total | 244±46 | 161±45 |  |
| Iodine, µg | Male | 384±635 | 115±61 | .117 |
|  | Female | 166±50 | 170±149 | .985 |
|  | Total | 275±449 | 142±114 |  |
| Selenium, µg | Male | 142±25 | 116±23 | .004 |
|  | Female | 95±20 | 96±21 | .863 |
|  | Total | 118±33 | 106±24 |  |
| Sodium, mg | Male | 1523±730 | 1354±866 | .614 |
|  | Female | 2144±1154 | 1515±618 | .076 |
|  | Total | 1833±986 | 1434±731 |  |
| Salt, mg | Male | 3824±1875 | 3450±2205 | .633 |
|  | Female | 5300±2719 | 3860±1574 | .082 |
|  | Total | 4562±2382 | 3655±1863 |  |
| Cholesterol, mg | Male | 792±255 | 566±174 | .002 |
|  | Female | 343±192 | 352±177 | .888 |
|  | Total | 567±318 | 459±203 |  |

**Table S3. 988 significantly differing features between the timepoints revealed by DeSeq2 analysis**

|  | FDR | Name | lFC with Error |
| --- | --- | --- | --- |
| K00690 | 4,1254E-06 | sucrose phosphorylase [EC:2.4.1.7] | -1,1+/-0,18 |
| K04488 | 3,6208E-05 | nitrogen fixation protein NifU and related proteins | -0,4+/-0,07 |
| K06320 | 0,00004364 | spore maturation protein CgeB | -1,15+/-0,21 |
| K01308 | 6,1838E-05 | g-D-glutamyl-meso-diaminopimelate peptidase [EC:3.4.19.11] | -0,93+/-0,17 |
| K15770 | 6,7197E-05 | arabinogalactan oligomer / maltooligosaccharide transport system substrate-binding protein | -0,77+/-0,14 |
| K01739 | 6,7197E-05 | cystathionine gamma-synthase [EC:2.5.1.48] | -0,76+/-0,14 |
| K15771 | 9,9621E-05 | arabinogalactan oligomer / maltooligosaccharide transport system permease protein | -0,75+/-0,14 |
| K11104 | 9,9621E-05 | melibiose permease | -0,57+/-0,11 |
| K03657 | 9,9621E-05 | ATP-dependent DNA helicase UvrD/PcrA [EC:5.6.2.4] | -0,22+/-0,04 |
| K10118 | 9,9621E-05 | raffinose/stachyose/melibiose transport system permease protein | -0,51+/-0,1 |
| K02647 | 9,9621E-05 | carbohydrate diacid regulator | -0,45+/-0,09 |
| K01421 | 9,9621E-05 | putative membrane protein | -0,5+/-0,1 |
| K03292 | 9,9621E-05 | glycoside/pentoside/hexuronide:cation symporter, GPH family | -0,36+/-0,07 |
| K19294 | 9,9621E-05 | alginate O-acetyltransferase complex protein AlgI | -0,47+/-0,09 |
| K10189 | 9,9621E-05 | lactose/L-arabinose transport system permease protein | -0,85+/-0,17 |
| K15772 | 9,9621E-05 | arabinogalactan oligomer / maltooligosaccharide transport system permease protein | -0,71+/-0,14 |
| K03320 | 9,9621E-05 | ammonium transporter, Amt family | -0,27+/-0,05 |
| K02438 | 9,9621E-05 | glycogen debranching enzyme [EC:3.2.1.196] | -0,6+/-0,12 |
| K10190 | 9,9621E-05 | lactose/L-arabinose transport system permease protein | -0,84+/-0,17 |
| K00375 | 9,9621E-05 | GntR family transcriptional regulator / MocR family aminotransferase | -0,38+/-0,07 |
| K05833 | 0,0001155 | putative tryptophan/tyrosine transport system ATP-binding protein | -0,42+/-0,09 |
| K03484 | 0,0001155 | LacI family transcriptional regulator, sucrose operon repressor | -0,55+/-0,11 |
| K02283 | 0,0001155 | pilus assembly protein CpaF [EC:7.4.2.8] | -0,53+/-0,11 |
| K05832 | 0,0001155 | putative tryptophan/tyrosine transport system permease protein | -0,42+/-0,08 |
| K02203 | 0,0001155 | phosphoserine / homoserine phosphotransferase [EC:3.1.3.3 2.7.1.39] | -0,56+/-0,11 |
| K15634 | 0,0001155 | 2,3-bisphosphoglycerate-dependent phosphoglycerate mutase [EC:5.4.2.11] | -0,39+/-0,08 |
| K10188 | 0,0001155 | lactose/L-arabinose transport system substrate-binding protein | -0,84+/-0,17 |
| K02072 | 0,0001155 | D-methionine transport system permease protein | -0,33+/-0,07 |
| K16841 | 0,0001155 | allantoin racemase [EC:5.1.99.3] | 1,47+/-0,3 |
| K10119 | 0,0001155 | raffinose/stachyose/melibiose transport system permease protein | -0,49+/-0,1 |
| K10117 | 0,00011779 | raffinose/stachyose/melibiose transport system substrate-binding protein | -0,44+/-0,09 |
| K08659 | 0,00012 | dipeptidase [EC:3.4.-.-] | -0,56+/-0,11 |
| K07749 | 0,00012432 | formyl-CoA transferase [EC:2.8.3.16] | 1,59+/-0,32 |
| K01652 | 0,00012432 | acetolactate synthase I/II/III large subunit [EC:2.2.1.6] | -0,18+/-0,04 |
| K16511 | 0,00012432 | adapter protein MecA 1/2 | -0,64+/-0,13 |
| K09759 | 0,00012432 | nondiscriminating aspartyl-tRNA synthetase [EC:6.1.1.23] | -0,71+/-0,14 |
| K00821 | 0,00013175 | acetylornithine/N-succinyldiaminopimelate aminotransferase [EC:2.6.1.11 2.6.1.17] | -0,43+/-0,09 |
| K06310 | 0,00013359 | spore germination protein | -0,76+/-0,16 |
| K00605 | 0,00013359 | glycine cleavage system T protein (aminomethyltransferase) [EC:2.1.2.10] | 0,46+/-0,1 |
| K11184 | 0,00013492 | catabolite repression HPr-like protein | -0,71+/-0,15 |
| K01193 | 0,0001403 | beta-fructofuranosidase [EC:3.2.1.26] | -0,49+/-0,1 |
| K02071 | 0,0001403 | D-methionine transport system ATP-binding protein | -0,32+/-0,07 |
| K01989 | 0,0001476 | putative tryptophan/tyrosine transport system substrate-binding protein | -0,4+/-0,08 |
| K11189 | 0,0001476 | uncharacterized | -0,38+/-0,08 |
| K05896 | 0,00015239 | segregation and condensation protein A | -0,35+/-0,07 |
| K07816 | 0,00015267 | GTP pyrophosphokinase [EC:2.7.6.5] | -0,57+/-0,12 |
| K06024 | 0,00015267 | segregation and condensation protein B | -0,35+/-0,07 |
| K06958 | 0,00015342 | RNase adapter protein RapZ | -0,33+/-0,07 |
| K06409 | 0,00015342 | stage V sporulation protein B | -0,43+/-0,09 |
| K00763 | 0,00015342 | nicotinate phosphoribosyltransferase [EC:6.3.4.21] | -0,27+/-0,06 |
| K03529 | 0,00016268 | chromosome segregation protein | -0,34+/-0,07 |
| K03523 | 0,00016268 | biotin transport system substrate-specific component | -0,38+/-0,08 |
| K07335 | 0,0001743 | basic membrane protein A and related proteins | -0,35+/-0,07 |
| K03763 | 0,00017612 | DNA polymerase III subunit alpha, Gram-positive type [EC:2.7.7.7] | -0,43+/-0,09 |
| K09989 | 0,00019335 | uncharacterized protein | 1,78+/-0,38 |
| K06907 | 0,00019377 | Bacteriophage tail sheath protein | 1,03+/-0,22 |
| K02030 | 0,00019377 | polar amino acid transport system substrate-binding protein | -0,3+/-0,06 |
| K03785 | 0,00019814 | 3-dehydroquinate dehydratase I [EC:4.2.1.10] | -0,49+/-0,1 |
| K01501 | 0,00020123 | nitrilase [EC:3.5.5.1] | 1,79+/-0,38 |
| K01486 | 0,00020191 | adenine deaminase [EC:3.5.4.2] | -0,43+/-0,09 |
| K03798 | 0,00020916 | cell division protease FtsH [EC:3.4.24.-] | -0,23+/-0,05 |
| K06147 | 0,00020916 | ATP-binding cassette, subfamily B, bacterial | -0,32+/-0,07 |
| K02406 | 0,00021919 | flagellin | -0,68+/-0,15 |
| K16899 | 0,00023192 | ATP-dependent helicase/nuclease subunit B [EC:5.6.2.4 3.1.-.-] | -0,38+/-0,08 |
| K03544 | 0,00023192 | ATP-dependent Clp protease ATP-binding subunit ClpX | -0,19+/-0,04 |
| K06923 | 0,00023192 | uncharacterized protein | -0,37+/-0,08 |
| K09697 | 0,00023192 | sodium transport system ATP-binding protein [EC:7.2.2.4] | -0,98+/-0,21 |
| K09696 | 0,00023192 | sodium transport system permease protein | -0,97+/-0,21 |
| K01567 | 0,00023192 | peptidoglycan-N-acetylmuramic acid deacetylase [EC:3.5.1.-] | -0,52+/-0,11 |
| K18968 | 0,00023525 | diguanylate cyclase [EC:2.7.7.65] | 1,54+/-0,33 |
| K02026 | 0,00023525 | multiple sugar transport system permease protein | -0,38+/-0,08 |
| K07007 | 0,00023525 | 3-dehydro-bile acid Delta4,6-reductase [EC:1.3.1.114] | -0,28+/-0,06 |
| K00606 | 0,00023525 | 3-methyl-2-oxobutanoate hydroxymethyltransferase [EC:2.1.2.11] | 0,42+/-0,09 |
| K15533 | 0,00023525 | 1,3-beta-galactosyl-N-acetylhexosamine phosphorylase [EC:2.4.1.211] | -0,45+/-0,1 |
| K05364 | 0,00023876 | penicillin-binding protein A | -0,54+/-0,12 |
| K04042 | 0,00024941 | bifunctional UDP-N-acetylglucosamine pyrophosphorylase / glucosamine-1-phosphate N-acetyltransferase [EC:2.7.7.23 2.3.1.157] | -0,32+/-0,07 |
| K10254 | 0,00025089 | oleate hydratase [EC:4.2.1.53] | -0,46+/-0,1 |
| K03608 | 0,00025324 | cell division topological specificity factor | -0,54+/-0,12 |
| K02437 | 0,00025324 | glycine cleavage system H protein | 0,42+/-0,09 |
| K01182 | 0,00025556 | oligo-1,6-glucosidase [EC:3.2.1.10] | -0,58+/-0,13 |
| K01029 | 0,00025556 | 3-oxoacid CoA-transferase subunit B [EC:2.8.3.5] | 1,68+/-0,37 |
| K02003 | 0,00027388 | putative ABC transport system ATP-binding protein | -0,28+/-0,06 |
| K09768 | 0,0002771 | uncharacterized protein | -0,42+/-0,09 |
| K06903 | 0,00027741 | Bacteriophage baseplate protein W | 1,02+/-0,23 |
| K03406 | 0,00028385 | methyl-accepting chemotaxis protein | -0,69+/-0,15 |
| K16898 | 0,00028385 | ATP-dependent helicase/nuclease subunit A [EC:5.6.2.4 3.1.-.-] | -0,36+/-0,08 |
| K00974 | 0,00029076 | tRNA nucleotidyltransferase (CCA-adding enzyme) [EC:2.7.7.72 3.1.3.- 3.1.4.-] | -0,32+/-0,07 |
| K01692 | 0,00029076 | enoyl-CoA hydratase [EC:4.2.1.17] | 1,92+/-0,42 |
| K12510 | 0,00029421 | tight adherence protein B | -0,48+/-0,11 |
| K05936 | 0,00029421 | precorrin-4/cobalt-precorrin-4 C11-methyltransferase [EC:2.1.1.133 2.1.1.271] | -0,42+/-0,09 |
| K03561 | 0,00030292 | biopolymer transport protein ExbB | 0,46+/-0,1 |
| K08884 | 0,00031571 | serine/threonine protein kinase, bacterial [EC:2.7.11.1] | -0,37+/-0,08 |
| K00655 | 0,00032652 | 1-acyl-sn-glycerol-3-phosphate acyltransferase [EC:2.3.1.51] | -0,19+/-0,04 |
| K08384 | 0,00032652 | stage V sporulation protein D (sporulation-specific penicillin-binding protein) | -0,43+/-0,09 |
| K03686 | 0,00032652 | molecular chaperone DnaJ | -0,23+/-0,05 |
| K09777 | 0,00032652 | extracellular matrix regulatory protein A | -0,42+/-0,09 |
| K06412 | 0,00034214 | stage V sporulation protein G | -0,49+/-0,11 |
| K09157 | 0,00034232 | uncharacterized protein | -0,35+/-0,08 |
| K03431 | 0,00034232 | phosphoglucosamine mutase [EC:5.4.2.10] | -0,35+/-0,08 |
| K02422 | 0,00034326 | flagellar secretion chaperone FliS | -0,65+/-0,15 |
| K03563 | 0,00034326 | carbon storage regulator | -0,65+/-0,15 |
| K03571 | 0,00034326 | rod shape-determining protein MreD | -0,43+/-0,1 |
| K10112 | 0,00034924 | multiple sugar transport system ATP-binding protein [EC:7.5.2.-] | -0,34+/-0,08 |
| K02945 | 0,00035185 | small subunit ribosomal protein S1 | -0,18+/-0,04 |
| K01653 | 0,00035185 | acetolactate synthase I/III small subunit [EC:2.2.1.6] | -0,19+/-0,04 |
| K12267 | 0,00035423 | peptide methionine sulfoxide reductase msrA/msrB [EC:1.8.4.11 1.8.4.12] | 0,39+/-0,09 |
| K02315 | 0,00035679 | DNA replication protein DnaC | -0,42+/-0,1 |
| K04487 | 0,0003766 | cysteine desulfurase [EC:2.8.1.7] | -0,33+/-0,08 |
| K03281 | 0,00037688 | chloride channel protein, CIC family | 0,54+/-0,12 |
| K16789 | 0,00038728 | thiamine transporter | -0,41+/-0,09 |
| K02502 | 0,00040061 | ATP phosphoribosyltransferase regulatory subunit | -0,35+/-0,08 |
| K16171 | 0,00043275 | fumarylacetoacetate (FAA) hydrolase [EC:3.7.1.2] | 2+/-0,46 |
| K02028 | 0,0004446 | polar amino acid transport system ATP-binding protein [EC:7.4.2.1] | -0,25+/-0,06 |
| K11051 | 0,0004446 | multidrug/hemolysin transport system permease protein | -0,79+/-0,18 |
| K11050 | 0,00044495 | multidrug/hemolysin transport system ATP-binding protein | -0,79+/-0,18 |
| K18475 | 0,00045522 | lysine-N-methylase [EC:2.1.1.-] | -0,56+/-0,13 |
| K04027 | 0,00045522 | ethanolamine utilization protein EutM | 1,75+/-0,4 |
| K03624 | 0,00046428 | transcription elongation factor GreA | -0,22+/-0,05 |
| K01847 | 0,00047023 | methylmalonyl-CoA mutase [EC:5.4.99.2] | 0,52+/-0,12 |
| K01963 | 0,00048691 | acetyl-CoA carboxylase carboxyl transferase subunit beta [EC:6.4.1.2 2.1.3.15] | -0,35+/-0,08 |
| K07502 | 0,00048691 | uncharacterized protein | -0,61+/-0,14 |
| K02407 | 0,00049353 | flagellar hook-associated protein 2 | -0,66+/-0,15 |
| K00428 | 0,00049353 | cytochrome c peroxidase [EC:1.11.1.5] | 1,32+/-0,31 |
| K03609 | 0,00049353 | septum site-determining protein MinD | -0,39+/-0,09 |
| K09779 | 0,00049415 | uncharacterized protein | -0,4+/-0,09 |
| K07166 | 0,00050636 | ACT domain-containing protein | -0,36+/-0,08 |
| K00965 | 0,00050636 | UDPglucose--hexose-1-phosphate uridylyltransferase [EC:2.7.7.12] | -0,39+/-0,09 |
| K05350 | 0,00050636 | beta-glucosidase [EC:3.2.1.21] | -0,48+/-0,11 |
| K04070 | 0,00050761 | putative pyruvate formate lyase activating enzyme [EC:1.97.1.4] | -0,4+/-0,09 |
| K15866 | 0,00050761 | 2-(1,2-epoxy-1,2-dihydrophenyl)acetyl-CoA isomerase [EC:5.3.3.18] | 1,48+/-0,34 |
| K16787 | 0,00050761 | energy-coupling factor transport system ATP-binding protein [EC:7.-.-.-] | -0,32+/-0,07 |
| K02372 | 0,00050761 | 3-hydroxyacyl-[acyl-carrier-protein] dehydratase [EC:4.2.1.59] | -0,32+/-0,08 |
| K16785 | 0,00050761 | energy-coupling factor transport system permease protein | -0,32+/-0,07 |
| K01251 | 0,00050761 | adenosylhomocysteinase [EC:3.13.2.1] | 0,52+/-0,12 |
| K02189 | 0,00050761 | cobalt-precorrin 5A hydrolase [EC:3.7.1.12] | -0,41+/-0,1 |
| K02302 | 0,00050761 | uroporphyrin-III C-methyltransferase / precorrin-2 dehydrogenase / sirohydrochlorin ferrochelatase [EC:2.1.1.107 1.3.1.76 4.99.1.4] | -0,62+/-0,14 |
| K07574 | 0,00050761 | RNA-binding protein | -0,36+/-0,08 |
| K07027 | 0,00050761 | glycosyltransferase 2 family protein | -0,33+/-0,08 |
| K01520 | 0,00050761 | dUTP diphosphatase [EC:3.6.1.23] | -0,27+/-0,06 |
| K03705 | 0,00051313 | heat-inducible transcriptional repressor | -0,34+/-0,08 |
| K11928 | 0,00051313 | sodium/proline symporter | -0,43+/-0,1 |
| K07720 | 0,00051499 | two-component system, response regulator YesN | -0,4+/-0,09 |
| K03091 | 0,00051499 | RNA polymerase sigma-E/F/G factor | -0,39+/-0,09 |
| K13052 | 0,00051854 | cell division protein DivIC | -0,44+/-0,1 |
| K06403 | 0,00052242 | stage V sporulation protein AA | -0,62+/-0,15 |
| K06404 | 0,00052242 | stage V sporulation protein AB | -0,62+/-0,15 |
| K05606 | 0,00053311 | methylmalonyl-CoA/ethylmalonyl-CoA epimerase [EC:5.1.99.1] | 0,48+/-0,11 |
| K00632 | 0,0005417 | acetyl-CoA acyltransferase [EC:2.3.1.16] | 1,44+/-0,34 |
| K01356 | 0,00056869 | repressor LexA [EC:3.4.21.88] | -0,36+/-0,08 |
| K07114 | 0,00057003 | Ca-activated chloride channel homolog | 0,44+/-0,1 |
| K10540 | 0,00058243 | methyl-galactoside transport system substrate-binding protein | -0,45+/-0,11 |
| K07035 | 0,00059424 | uncharacterized protein | -0,47+/-0,11 |
| K00016 | 0,00062002 | L-lactate dehydrogenase [EC:1.1.1.27] | -0,47+/-0,11 |
| K07588 | 0,0006216 | GTPase [EC:3.6.5.-] | 0,48+/-0,11 |
| K00882 | 0,0006216 | 1-phosphofructokinase [EC:2.7.1.56] | -0,37+/-0,09 |
| K02022 | 0,0006216 | HlyD family secretion protein | 1,2+/-0,28 |
| K09762 | 0,0006216 | cell division protein WhiA | -0,36+/-0,09 |
| K02396 | 0,00062206 | flagellar hook-associated protein 1 | -0,63+/-0,15 |
| K01838 | 0,0006368 | beta-phosphoglucomutase [EC:5.4.2.6] | -0,73+/-0,17 |
| K14761 | 0,0006368 | ribosome-associated protein | -0,37+/-0,09 |
| K00797 | 0,0006368 | spermidine synthase [EC:2.5.1.16] | -0,42+/-0,1 |
| K06173 | 0,0006368 | tRNA pseudouridine38-40 synthase [EC:5.4.99.12] | -0,17+/-0,04 |
| K03458 | 0,00065647 | nucleobase:cation symporter-2, NCS2 family | -0,39+/-0,09 |
| K06182 | 0,00067222 | 23S rRNA pseudouridine2604 synthase [EC:5.4.99.21] | -0,49+/-0,12 |
| K06967 | 0,0006746 | tRNA (adenine22-N1)-methyltransferase [EC:2.1.1.217] | -0,36+/-0,08 |
| K00641 | 0,0006746 | homoserine O-acetyltransferase/O-succinyltransferase [EC:2.3.1.31 2.3.1.46] | 0,91+/-0,22 |
| K00975 | 0,0006746 | glucose-1-phosphate adenylyltransferase [EC:2.7.7.27] | -0,38+/-0,09 |
| K06200 | 0,00068812 | carbon starvation protein | -0,41+/-0,1 |
| K02278 | 0,0006901 | prepilin peptidase CpaA [EC:3.4.23.43] | -0,54+/-0,13 |
| K03559 | 0,00070725 | biopolymer transport protein ExbD | 0,58+/-0,14 |
| K01338 | 0,00071565 | ATP-dependent Lon protease [EC:3.4.21.53] | -0,21+/-0,05 |
| K02025 | 0,00071565 | multiple sugar transport system permease protein | -0,35+/-0,08 |
| K08483 | 0,00072272 | phosphoenolpyruvate-protein phosphotransferase (PTS system enzyme I) [EC:2.7.3.9] | -0,3+/-0,07 |
| K07173 | 0,00073963 | S-ribosylhomocysteine lyase [EC:4.4.1.21] | -0,39+/-0,09 |
| K07979 | 0,00074379 | GntR family transcriptional regulator | -0,37+/-0,09 |
| K09772 | 0,00074379 | cell division inhibitor SepF | -0,35+/-0,09 |
| K01918 | 0,00074379 | pantoate--beta-alanine ligase [EC:6.3.2.1] | 0,37+/-0,09 |
| K07105 | 0,00076712 | uncharacterized protein | -0,4+/-0,1 |
| K17734 | 0,00081238 | serine protease AprX [EC:3.4.21.-] | -0,59+/-0,14 |
| K07738 | 0,0008166 | transcriptional repressor NrdR | -0,26+/-0,06 |
| K06382 | 0,0008166 | stage II sporulation protein E [EC:3.1.3.16] | -0,4+/-0,1 |
| K14540 | 0,00085635 | ribosome biogenesis GTPase A | -0,34+/-0,08 |
| K04769 | 0,00085635 | AbrB family transcriptional regulator, stage V sporulation protein T | -0,39+/-0,1 |
| K06381 | 0,00086109 | stage II sporulation protein D | -0,4+/-0,1 |
| K00820 | 0,00086109 | glutamine---fructose-6-phosphate transaminase (isomerizing) [EC:2.6.1.16] | -0,18+/-0,04 |
| K13688 | 0,00086109 | cyclic beta-1,2-glucan synthetase [EC:2.4.1.-] | 1,67+/-0,4 |
| K06283 | 0,00086109 | putative DeoR family transcriptional regulator, stage III sporulation protein D | -0,4+/-0,1 |
| K13963 | 0,00086109 | serpin B | -0,74+/-0,18 |
| K19221 | 0,00086109 | cob(I)alamin adenosyltransferase [EC:2.5.1.17] | -0,36+/-0,09 |
| K06396 | 0,00086109 | stage III sporulation protein AG | -0,4+/-0,1 |
| K06397 | 0,00086109 | stage III sporulation protein AH | -0,4+/-0,1 |
| K06392 | 0,00087013 | stage III sporulation protein AC | -0,4+/-0,1 |
| K06390 | 0,00087301 | stage III sporulation protein AA | -0,4+/-0,1 |
| K06393 | 0,00087301 | stage III sporulation protein AD | -0,4+/-0,1 |
| K03637 | 0,00090819 | cyclic pyranopterin monophosphate synthase [EC:4.6.1.17] | -0,39+/-0,09 |
| K05800 | 0,00090819 | Lrp/AsnC family transcriptional regulator, cysteine-sensing transcriptional activator | 1,14+/-0,28 |
| K03101 | 0,00091095 | signal peptidase II [EC:3.4.23.36] | -0,16+/-0,04 |
| K03639 | 0,00093953 | GTP 3',8-cyclase [EC:4.1.99.22] | -0,36+/-0,09 |
| K07258 | 0,00096078 | serine-type D-Ala-D-Ala carboxypeptidase (penicillin-binding protein 5/6) [EC:3.4.16.4] | -0,35+/-0,09 |
| K03410 | 0,00098932 | chemotaxis protein CheC | -0,65+/-0,16 |
| K01677 | 0,0010032 | fumarate hydratase subunit alpha [EC:4.2.1.2] | -0,35+/-0,09 |
| K03438 | 0,0010032 | 16S rRNA (cytosine1402-N4)-methyltransferase [EC:2.1.1.199] | -0,17+/-0,04 |
| K01839 | 0,0010048 | phosphopentomutase [EC:5.4.2.7] | -0,39+/-0,09 |
| K01678 | 0,0010344 | fumarate hydratase subunit beta [EC:4.2.1.2] | -0,36+/-0,09 |
| K12992 | 0,0010466 | O-antigen biosynthesis alpha-1,3-rhamnosyltransferase [EC:2.4.1.377] | -0,65+/-0,16 |
| K03205 | 0,0010466 | type IV secretion system protein VirD4 [EC:7.4.2.8] | -0,36+/-0,09 |
| K03585 | 0,0010466 | membrane fusion protein, multidrug efflux system | 0,38+/-0,09 |
| K02237 | 0,0010694 | competence protein ComEA | -0,37+/-0,09 |
| K01512 | 0,0010697 | acylphosphatase [EC:3.6.1.7] | -0,45+/-0,11 |
| K02470 | 0,0010908 | DNA gyrase subunit B [EC:5.6.2.2] | -0,19+/-0,05 |
| K07171 | 0,001113 | mRNA interferase MazF [EC:3.1.-.-] | -0,36+/-0,09 |
| K01579 | 0,0011146 | aspartate 1-decarboxylase [EC:4.1.1.11] | 0,36+/-0,09 |
| K09787 | 0,0011194 | uncharacterized protein | -0,36+/-0,09 |
| K05934 | 0,0011433 | precorrin-3B C17-methyltransferase / cobalt-factor III methyltransferase [EC:2.1.1.131 2.1.1.272] | -0,39+/-0,1 |
| K02113 | 0,0011522 | F-type H+-transporting ATPase subunit delta | -0,25+/-0,06 |
| K06042 | 0,0011522 | precorrin-8X/cobalt-precorrin-8 methylmutase [EC:5.4.99.61 5.4.99.60] | -0,35+/-0,09 |
| K03151 | 0,0011701 | tRNA uracil 4-sulfurtransferase [EC:2.8.1.4] | -0,34+/-0,09 |
| K06398 | 0,0011817 | stage IV sporulation protein A | -0,38+/-0,09 |
| K06001 | 0,0011913 | tryptophan synthase beta chain [EC:4.2.1.20] | 0,44+/-0,11 |
| K11145 | 0,0011913 | mini-ribonuclease III [EC:3.1.26.-] | -0,36+/-0,09 |
| K03474 | 0,0011913 | pyridoxine 5-phosphate synthase [EC:2.6.99.2] | 0,41+/-0,1 |
| K00756 | 0,0011921 | pyrimidine-nucleoside phosphorylase [EC:2.4.2.2] | -0,41+/-0,1 |
| K18828 | 0,0012045 | tRNA(fMet)-specific endonuclease VapC [EC:3.1.-.-] | 0,88+/-0,22 |
| K19092 | 0,0012282 | toxin ParE1/3/4 | -0,84+/-0,21 |
| K01299 | 0,0012282 | carboxypeptidase Taq [EC:3.4.17.19] | -0,74+/-0,18 |
| K06400 | 0,0012304 | site-specific DNA recombinase | -0,4+/-0,1 |
| K02056 | 0,0012304 | simple sugar transport system ATP-binding protein [EC:7.5.2.-] | -0,26+/-0,07 |
| K07282 | 0,0012304 | gamma-polyglutamate biosynthesis protein CapA | -0,24+/-0,06 |
| K02027 | 0,0012334 | multiple sugar transport system substrate-binding protein | -0,32+/-0,08 |
| K00040 | 0,0012382 | fructuronate reductase [EC:1.1.1.57] | -0,48+/-0,12 |
| K06901 | 0,0012429 | adenine/guanine/hypoxanthine permease | -0,32+/-0,08 |
| K06167 | 0,0013101 | phosphoribosyl 1,2-cyclic phosphate phosphodiesterase [EC:3.1.4.55] | 0,39+/-0,1 |
| K13292 | 0,0013101 | phosphatidylglycerol---prolipoprotein diacylglyceryl transferase [EC:2.5.1.145] | -0,26+/-0,07 |
| K02415 | 0,001355 | flagellar protein FliL | -0,82+/-0,21 |
| K06399 | 0,001355 | stage IV sporulation protein B [EC:3.4.21.116] | -0,37+/-0,09 |
| K03496 | 0,0013634 | chromosome partitioning protein | -0,21+/-0,05 |
| K06379 | 0,001372 | stage II sporulation protein AB (anti-sigma F factor) [EC:2.7.11.1] | -0,37+/-0,09 |
| K07742 | 0,0013753 | uncharacterized protein | -0,35+/-0,09 |
| K06378 | 0,001381 | stage II sporulation protein AA (anti-sigma F factor antagonist) | -0,37+/-0,09 |
| K01960 | 0,001381 | pyruvate carboxylase subunit B [EC:6.4.1.1] | 0,4+/-0,1 |
| K06385 | 0,0013818 | stage II sporulation protein P | -0,38+/-0,1 |
| K06405 | 0,0014181 | stage V sporulation protein AC | -0,37+/-0,09 |
| K06012 | 0,0014235 | spore protease [EC:3.4.24.78] | -0,37+/-0,09 |
| K00240 | 0,001436 | succinate dehydrogenase iron-sulfur subunit [EC:1.3.5.1] | 0,42+/-0,11 |
| K06406 | 0,0014409 | stage V sporulation protein AD | -0,37+/-0,09 |
| K02469 | 0,0014409 | DNA gyrase subunit A [EC:5.6.2.2] | -0,19+/-0,05 |
| K00241 | 0,0014409 | succinate dehydrogenase cytochrome b subunit | 0,46+/-0,12 |
| K02392 | 0,0014477 | flagellar basal-body rod protein FlgG | -0,55+/-0,14 |
| K11754 | 0,0014501 | dihydrofolate synthase / folylpolyglutamate synthase [EC:6.3.2.12 6.3.2.17] | -0,2+/-0,05 |
| K06333 | 0,001468 | spore coat protein JB | -0,42+/-0,11 |
| K06153 | 0,0014799 | undecaprenyl-diphosphatase [EC:3.6.1.27] | -0,17+/-0,04 |
| K00060 | 0,0014799 | threonine 3-dehydrogenase [EC:1.1.1.103] | 1,33+/-0,34 |
| K00798 | 0,0014817 | cob(I)alamin adenosyltransferase [EC:2.5.1.17] | 0,46+/-0,12 |
| K06407 | 0,0014817 | stage V sporulation protein AE | -0,37+/-0,09 |
| K03700 | 0,0014989 | recombination protein U | -0,5+/-0,13 |
| K07533 | 0,0015133 | foldase protein PrsA [EC:5.2.1.8] | -0,54+/-0,14 |
| K07718 | 0,0015225 | two-component system, sensor histidine kinase YesM [EC:2.7.13.3] | -0,38+/-0,1 |
| K03310 | 0,0015324 | alanine or glycine:cation symporter, AGCS family | -0,29+/-0,07 |
| K03733 | 0,0015485 | integrase/recombinase XerC | 0,38+/-0,1 |
| K11358 | 0,0015929 | aspartate aminotransferase [EC:2.6.1.1] | -0,41+/-0,11 |
| K00970 | 0,0016036 | poly(A) polymerase [EC:2.7.7.19] | 0,37+/-0,1 |
| K00968 | 0,0016347 | choline-phosphate cytidylyltransferase [EC:2.7.7.15] | 1,3+/-0,33 |
| K01176 | 0,0016541 | alpha-amylase [EC:3.2.1.1] | 0,58+/-0,15 |
| K01816 | 0,0016541 | hydroxypyruvate isomerase [EC:5.3.1.22] | 0,89+/-0,23 |
| K02110 | 0,0016541 | F-type H+-transporting ATPase subunit c | -0,26+/-0,07 |
| K18907 | 0,0016571 | GntR family transcriptional regulator, regulator for abcA and norABC | 1,68+/-0,43 |
| K07015 | 0,0016617 | putative phosphatase [EC:3.1.3.-] | -0,37+/-0,1 |
| K16363 | 0,0016617 | UDP-3-O-[3-hydroxymyristoyl] N-acetylglucosamine deacetylase / 3-hydroxyacyl-[acyl-carrier-protein] dehydratase [EC:3.5.1.108 4.2.1.59] | 0,45+/-0,12 |
| K02529 | 0,001673 | LacI family transcriptional regulator | -0,24+/-0,06 |
| K07030 | 0,0016736 | fatty acid kinase [EC:2.7.2.18] | -0,35+/-0,09 |
| K17073 | 0,0016777 | putative lysine transport system substrate-binding protein | -0,66+/-0,17 |
| K00219 | 0,001678 | 2,4-dienoyl-CoA reductase (NADPH2) [EC:1.3.1.34] | 1,18+/-0,3 |
| K03789 | 0,0016843 | [ribosomal protein S18]-alanine N-acetyltransferase [EC:2.3.1.266] | -0,33+/-0,08 |
| K07658 | 0,001691 | two-component system, OmpR family, alkaline phosphatase synthesis response regulator PhoP | -0,24+/-0,06 |
| K06179 | 0,0017 | 23S rRNA pseudouridine955/2504/2580 synthase [EC:5.4.99.24] | -0,33+/-0,08 |
| K02224 | 0,0017148 | cobyrinic acid a,c-diamide synthase [EC:6.3.5.9 6.3.5.11] | -0,28+/-0,07 |
| K14393 | 0,0017148 | cation/acetate symporter | 2,13+/-0,55 |
| K02115 | 0,0017195 | F-type H+-transporting ATPase subunit gamma | -0,26+/-0,07 |
| K02426 | 0,0017195 | cysteine desulfuration protein SufE | 0,47+/-0,12 |
| K00027 | 0,00175 | malate dehydrogenase (oxaloacetate-decarboxylating) [EC:1.1.1.38] | -0,33+/-0,09 |
| K07164 | 0,0017519 | uncharacterized protein | 0,46+/-0,12 |
| K01243 | 0,0017519 | adenosylhomocysteine nucleosidase [EC:3.2.2.9] | -0,26+/-0,07 |
| K01854 | 0,0017521 | UDP-galactopyranose mutase [EC:5.4.99.9] | -0,36+/-0,09 |
| K03308 | 0,0017857 | neurotransmitter:Na+ symporter, NSS family | -0,25+/-0,06 |
| K04564 | 0,0018798 | superoxide dismutase, Fe-Mn family [EC:1.15.1.1] | 0,4+/-0,1 |
| K06334 | 0,0018819 | spore coat protein JC | -0,42+/-0,11 |
| K02017 | 0,001889 | molybdate transport system ATP-binding protein [EC:7.3.2.5] | -0,34+/-0,09 |
| K03581 | 0,0018983 | exodeoxyribonuclease V alpha subunit [EC:3.1.11.5] | -0,21+/-0,06 |
| K06209 | 0,0018983 | chorismate mutase [EC:5.4.99.5] | -0,38+/-0,1 |
| K06972 | 0,0019306 | presequence protease [EC:3.4.24.-] | -0,4+/-0,1 |
| K02824 | 0,0019344 | uracil permease | -0,28+/-0,07 |
| K19119 | 0,0019398 | CRISPR-associated protein Cas5d | 0,74+/-0,19 |
| K02111 | 0,0019405 | F-type H+/Na+-transporting ATPase subunit alpha [EC:7.1.2.2 7.2.2.1] | -0,26+/-0,07 |
| K07699 | 0,0019405 | two-component system, response regulator, stage 0 sporulation protein A | -0,35+/-0,09 |
| K07491 | 0,0019747 | REP-associated tyrosine transposase | -0,46+/-0,12 |
| K01990 | 0,0019747 | ABC-2 type transport system ATP-binding protein | -0,22+/-0,06 |
| K02108 | 0,0020055 | F-type H+-transporting ATPase subunit a | -0,26+/-0,07 |
| K01548 | 0,0020055 | potassium-transporting ATPase KdpC subunit | 0,47+/-0,12 |
| K02112 | 0,0020143 | F-type H+/Na+-transporting ATPase subunit beta [EC:7.1.2.2 7.2.2.1] | -0,26+/-0,07 |
| K01546 | 0,0020143 | potassium-transporting ATPase potassium-binding subunit | 0,47+/-0,12 |
| K06895 | 0,0020143 | L-lysine exporter family protein LysE/ArgO | 1,2+/-0,32 |
| K02114 | 0,0020226 | F-type H+-transporting ATPase subunit epsilon | -0,26+/-0,07 |
| K02527 | 0,0020226 | 3-deoxy-D-manno-octulosonic-acid transferase [EC:2.4.99.12 2.4.99.13 2.4.99.14 2.4.99.15] | 0,37+/-0,1 |
| K02057 | 0,0020226 | simple sugar transport system permease protein | -0,25+/-0,07 |
| K03215 | 0,0020226 | 23S rRNA (uracil1939-C5)-methyltransferase [EC:2.1.1.190] | -0,2+/-0,05 |
| K16786 | 0,0020226 | energy-coupling factor transport system ATP-binding protein [EC:7.-.-.-] | -0,34+/-0,09 |
| K04517 | 0,0020459 | prephenate dehydrogenase [EC:1.3.1.12] | -0,21+/-0,05 |
| K02495 | 0,0020459 | oxygen-independent coproporphyrinogen III oxidase [EC:1.3.98.3] | -0,17+/-0,05 |
| K00762 | 0,0020463 | orotate phosphoribosyltransferase [EC:2.4.2.10] | -0,19+/-0,05 |
| K01547 | 0,0020467 | potassium-transporting ATPase ATP-binding subunit [EC:7.2.2.6] | 0,47+/-0,12 |
| K03497 | 0,0020605 | ParB family transcriptional regulator, chromosome partitioning protein | -0,22+/-0,06 |
| K00805 | 0,0020605 | heptaprenyl diphosphate synthase component 1 [EC:2.5.1.30] | -0,34+/-0,09 |
| K00533 | 0,0020605 | ferredoxin hydrogenase large subunit [EC:1.12.7.2] | -0,58+/-0,15 |
| K07405 | 0,002064 | alpha-amylase [EC:3.2.1.1] | 0,45+/-0,12 |
| K02109 | 0,0021015 | F-type H+-transporting ATPase subunit b | -0,25+/-0,07 |
| K03179 | 0,0021911 | 4-hydroxybenzoate polyprenyltransferase [EC:2.5.1.39] | 0,85+/-0,22 |
| K03546 | 0,002291 | DNA repair protein SbcC/Rad50 | -0,3+/-0,08 |
| K03709 | 0,002291 | DtxR family transcriptional regulator, Mn-dependent transcriptional regulator | 0,95+/-0,25 |
| K12574 | 0,0023191 | ribonuclease J [EC:3.1.-.-] | -0,32+/-0,09 |
| K09747 | 0,0023294 | nucleoid-associated protein EbfC | -0,24+/-0,06 |
| K00284 | 0,0023296 | glutamate synthase (ferredoxin) [EC:1.4.7.1] | -0,39+/-0,1 |
| K06133 | 0,0023562 | 4'-phosphopantetheinyl transferase [EC:2.7.8.-] | -0,44+/-0,12 |
| K06394 | 0,0024528 | stage III sporulation protein AE | -0,43+/-0,11 |
| K01654 | 0,0024528 | N-acetylneuraminate synthase [EC:2.5.1.56] | -0,63+/-0,17 |
| K13051 | 0,0024559 | L-asparaginase / beta-aspartyl-peptidase [EC:3.5.1.1 3.4.19.5] | 0,93+/-0,25 |
| K01267 | 0,0024866 | aspartyl aminopeptidase [EC:3.4.11.21] | -0,35+/-0,09 |
| K01626 | 0,0024866 | 3-deoxy-7-phosphoheptulonate synthase [EC:2.5.1.54] | -0,31+/-0,08 |
| K04751 | 0,0024916 | nitrogen regulatory protein P-II 1 | -0,37+/-0,1 |
| K03826 | 0,0025015 | putative acetyltransferase [EC:2.3.1.-] | -0,46+/-0,12 |
| K02507 | 0,0025015 | protein transport protein HofQ | -3,03+/-0,81 |
| K07052 | 0,0025091 | CAAX protease family protein | -0,21+/-0,06 |
| K06438 | 0,0025091 | similar to stage IV sporulation protein | -0,42+/-0,11 |
| K03327 | 0,0025582 | MATE family, multidrug and toxin extrusion protein | 0,34+/-0,09 |
| K02188 | 0,0025679 | cobalt-precorrin-5B (C1)-methyltransferase [EC:2.1.1.195] | -0,32+/-0,09 |
| K07584 | 0,0025762 | uncharacterized protein | -0,41+/-0,11 |
| K00266 | 0,0026168 | glutamate synthase (NADPH) small chain [EC:1.4.1.13] | -0,21+/-0,06 |
| K07120 | 0,0026168 | uncharacterized protein | 1,21+/-0,32 |
| K07284 | 0,0026405 | sortase A [EC:3.4.22.70] | -0,4+/-0,11 |
| K00130 | 0,0026405 | betaine-aldehyde dehydrogenase [EC:1.2.1.8] | 2,12+/-0,57 |
| K01560 | 0,0026564 | 2-haloacid dehalogenase [EC:3.8.1.2] | -0,35+/-0,09 |
| K00759 | 0,0027081 | adenine phosphoribosyltransferase [EC:2.4.2.7] | -0,28+/-0,08 |
| K02018 | 0,0027922 | molybdate transport system permease protein | -0,32+/-0,09 |
| K03402 | 0,0028145 | transcriptional regulator of arginine metabolism | -0,27+/-0,07 |
| K07444 | 0,0028145 | putative N6-adenine-specific DNA methylase [EC:2.1.1.-] | -0,27+/-0,07 |
| K06387 | 0,0028265 | stage II sporulation protein R | -0,42+/-0,12 |
| K00703 | 0,0028283 | starch synthase [EC:2.4.1.21] | -0,2+/-0,06 |
| K02014 | 0,0028367 | iron complex outermembrane recepter protein | 0,38+/-0,1 |
| K00526 | 0,00286 | ribonucleoside-diphosphate reductase beta chain [EC:1.17.4.1] | -0,3+/-0,08 |
| K03710 | 0,0028618 | GntR family transcriptional regulator | -0,18+/-0,05 |
| K11072 | 0,0028618 | spermidine/putrescine transport system ATP-binding protein [EC:7.6.2.11] | -0,27+/-0,07 |
| K07141 | 0,0028618 | molybdenum cofactor cytidylyltransferase [EC:2.7.7.76] | -0,37+/-0,1 |
| K00337 | 0,0028764 | NADH-quinone oxidoreductase subunit H [EC:7.1.1.2] | 0,47+/-0,13 |
| K07183 | 0,0028774 | two-component system, response regulator / RNA-binding antiterminator | -0,31+/-0,08 |
| K03821 | 0,0028774 | poly[(R)-3-hydroxyalkanoate] polymerase subunit PhaC [EC:2.3.1.304] | 2,19+/-0,6 |
| K01737 | 0,0028827 | 6-pyruvoyltetrahydropterin/6-carboxytetrahydropterin synthase [EC:4.2.3.12 4.1.2.50] | 0,29+/-0,08 |
| K07404 | 0,0030452 | 6-phosphogluconolactonase [EC:3.1.1.31] | -0,41+/-0,11 |
| K07318 | 0,0030568 | adenine-specific DNA-methyltransferase [EC:2.1.1.72] | 1,36+/-0,37 |
| K07657 | 0,003102 | two-component system, OmpR family, phosphate regulon response regulator PhoB | 0,56+/-0,15 |
| K19449 | 0,0031212 | XRE family transcriptional regulator, master regulator for biofilm formation | 1,66+/-0,46 |
| K00702 | 0,0031212 | cellobiose phosphorylase [EC:2.4.1.20] | -0,43+/-0,12 |
| K00257 | 0,0031212 | acyl-ACP dehydrogenase [EC:1.3.99.-] | 0,56+/-0,15 |
| K02757 | 0,003128 | beta-glucoside PTS system EIICBA component [EC:2.7.1.-] | -0,42+/-0,12 |
| K06905 | 0,0031537 | Bacteriophage probable baseplate hub protein | 1,32+/-0,36 |
| K00705 | 0,0031545 | 4-alpha-glucanotransferase [EC:2.4.1.25] | -0,28+/-0,08 |
| K07089 | 0,0031897 | uncharacterized protein | -0,32+/-0,09 |
| K07216 | 0,0032321 | hemerythrin | -0,49+/-0,13 |
| K00018 | 0,0032546 | glycerate dehydrogenase [EC:1.1.1.29] | -0,2+/-0,05 |
| K14170 | 0,0032617 | chorismate mutase / prephenate dehydratase [EC:5.4.99.5 4.2.1.51] | -0,24+/-0,07 |
| K00140 | 0,0033221 | malonate-semialdehyde dehydrogenase (acetylating) / methylmalonate-semialdehyde dehydrogenase [EC:1.2.1.18 1.2.1.27] | 1,3+/-0,36 |
| K06908 | 0,003325 | Bacteriophage tail tube protein | 1,32+/-0,36 |
| K07473 | 0,0033779 | DNA-damage-inducible protein J | -0,37+/-0,1 |
| K00382 | 0,0033798 | dihydrolipoyl dehydrogenase [EC:1.8.1.4] | 0,29+/-0,08 |
| K02106 | 0,0033816 | short-chain fatty acids transporter | 1,41+/-0,39 |
| K15977 | 0,0034114 | putative oxidoreductase | 0,69+/-0,19 |
| K01008 | 0,003428 | selenide, water dikinase [EC:2.7.9.3] | -0,36+/-0,1 |
| K07042 | 0,0034368 | probable rRNA maturation factor | -0,23+/-0,06 |
| K07794 | 0,0035029 | putative tricarboxylic transport membrane protein | 1,21+/-0,33 |
| K02020 | 0,0035208 | molybdate transport system substrate-binding protein | -0,31+/-0,09 |
| K02770 | 0,0035402 | fructose PTS system EIIBC or EIIC component [EC:2.7.1.202] | -0,38+/-0,11 |
| K03500 | 0,003626 | 16S rRNA (cytosine967-C5)-methyltransferase [EC:2.1.1.176] | -0,22+/-0,06 |
| K00946 | 0,0037041 | thiamine-monophosphate kinase [EC:2.7.4.16] | 0,39+/-0,11 |
| K04061 | 0,0037073 | flagellar biosynthesis protein | -0,51+/-0,14 |
| K09693 | 0,0037654 | teichoic acid transport system ATP-binding protein [EC:7.5.2.4] | -0,52+/-0,14 |
| K03711 | 0,003889 | Fur family transcriptional regulator, ferric uptake regulator | -0,22+/-0,06 |
| K00331 | 0,0040034 | NADH-quinone oxidoreductase subunit B [EC:7.1.1.2] | 0,46+/-0,13 |
| K03823 | 0,004039 | phosphinothricin acetyltransferase [EC:2.3.1.183] | -0,21+/-0,06 |
| K09692 | 0,0040439 | teichoic acid transport system permease protein | -0,54+/-0,15 |
| K03216 | 0,0040665 | tRNA (cytidine/uridine-2'-O-)-methyltransferase [EC:2.1.1.207] | -0,23+/-0,06 |
| K00339 | 0,0041079 | NADH-quinone oxidoreductase subunit J [EC:7.1.1.2] | 0,46+/-0,13 |
| K00340 | 0,0041079 | NADH-quinone oxidoreductase subunit K [EC:7.1.1.2] | 0,46+/-0,13 |
| K00343 | 0,0041079 | NADH-quinone oxidoreductase subunit N [EC:7.1.1.2] | 0,46+/-0,13 |
| K00342 | 0,0041373 | NADH-quinone oxidoreductase subunit M [EC:7.1.1.2] | 0,46+/-0,13 |
| K06131 | 0,0041482 | cardiolipin synthase A/B [EC:2.7.8.-] | -0,18+/-0,05 |
| K06298 | 0,0041829 | germination protein M | -0,46+/-0,13 |
| K00684 | 0,0042056 | leucyl/phenylalanyl-tRNA---protein transferase [EC:2.3.2.6] | 1,56+/-0,44 |
| K00338 | 0,0042294 | NADH-quinone oxidoreductase subunit I [EC:7.1.1.2] | 0,46+/-0,13 |
| K04083 | 0,0042294 | molecular chaperone Hsp33 | -0,24+/-0,07 |
| K00179 | 0,0042294 | indolepyruvate ferredoxin oxidoreductase, alpha subunit [EC:1.2.7.8] | 0,35+/-0,1 |
| K03100 | 0,0042682 | signal peptidase I [EC:3.4.21.89] | -0,18+/-0,05 |
| K17247 | 0,0042682 | methionine sulfoxide reductase heme-binding subunit | 2,05+/-0,58 |
| K00878 | 0,004286 | hydroxyethylthiazole kinase [EC:2.7.1.50] | -0,26+/-0,07 |
| K03735 | 0,004286 | ethanolamine ammonia-lyase large subunit [EC:4.3.1.7] | 1,1+/-0,31 |
| K09949 | 0,004286 | UDP-2,3-diacylglucosamine hydrolase [EC:3.6.1.54] | 1,13+/-0,32 |
| K09922 | 0,0043035 | uncharacterized protein | 0,36+/-0,1 |
| K00024 | 0,0043035 | malate dehydrogenase [EC:1.1.1.37] | 0,35+/-0,1 |
| K03736 | 0,0043189 | ethanolamine ammonia-lyase small subunit [EC:4.3.1.7] | 1,1+/-0,31 |
| K00341 | 0,0043226 | NADH-quinone oxidoreductase subunit L [EC:7.1.1.2] | 0,45+/-0,13 |
| K00180 | 0,0043226 | indolepyruvate ferredoxin oxidoreductase, beta subunit [EC:1.2.7.8] | 0,34+/-0,1 |
| K06383 | 0,0043469 | stage II sporulation protein GA (sporulation sigma-E factor processing peptidase) [EC:3.4.23.-] | -0,41+/-0,12 |
| K12373 | 0,0043921 | hexosaminidase [EC:3.2.1.52] | 0,48+/-0,14 |
| K06373 | 0,0045116 | spore maturation protein A | -0,47+/-0,13 |
| K06911 | 0,0045397 | quercetin 2,3-dioxygenase [EC:1.13.11.24] | 0,47+/-0,13 |
| K03324 | 0,0045397 | phosphate:Na+ symporter | -0,28+/-0,08 |
| K00318 | 0,0045532 | proline dehydrogenase [EC:1.5.5.2] | 1,02+/-0,29 |
| K10542 | 0,0045532 | methyl-galactoside transport system ATP-binding protein [EC:7.5.2.11] | -0,4+/-0,11 |
| K07403 | 0,0045557 | membrane-bound serine protease (ClpP class) | 0,97+/-0,28 |
| K08321 | 0,0045985 | 3-hydroxy-5-phosphonooxypentane-2,4-dione thiolase [EC:2.3.1.245] | 0,84+/-0,24 |
| K04026 | 0,0046084 | ethanolamine utilization protein EutL | 1,18+/-0,34 |
| K03642 | 0,0046418 | rare lipoprotein A | 0,77+/-0,22 |
| K01928 | 0,0046485 | UDP-N-acetylmuramoyl-L-alanyl-D-glutamate--2,6-diaminopimelate ligase [EC:6.3.2.13] | -0,15+/-0,04 |
| K00639 | 0,0047767 | glycine C-acetyltransferase [EC:2.3.1.29] | 0,5+/-0,14 |
| K10541 | 0,0048345 | methyl-galactoside transport system permease protein | -0,39+/-0,11 |
| K00372 | 0,0048345 | assimilatory nitrate reductase catalytic subunit [EC:1.7.99.-] | 1,89+/-0,54 |
| K03590 | 0,0048402 | cell division protein FtsA | 0,33+/-0,1 |
| K02067 | 0,0049082 | phospholipid/cholesterol/gamma-HCH transport system substrate-binding protein | 0,4+/-0,12 |
| K03502 | 0,0049377 | DNA polymerase V | -0,26+/-0,08 |
| K15987 | 0,0049537 | K(+)-stimulated pyrophosphate-energized sodium pump [EC:7.2.3.1] | 0,28+/-0,08 |
| K01991 | 0,0049583 | polysaccharide biosynthesis/export protein | 0,37+/-0,11 |
| K03820 | 0,0049628 | apolipoprotein N-acyltransferase [EC:2.3.1.269] | 0,56+/-0,16 |
| K01585 | 0,005021 | arginine decarboxylase [EC:4.1.1.19] | 0,43+/-0,12 |
| K03816 | 0,005021 | xanthine phosphoribosyltransferase [EC:2.4.2.22] | -0,25+/-0,07 |
| K09796 | 0,0050296 | periplasmic copper chaperone A | 2,23+/-0,64 |
| K11085 | 0,0050656 | ATP-binding cassette, subfamily B, bacterial MsbA [EC:7.5.2.6] | 0,38+/-0,11 |
| K07090 | 0,0050819 | uncharacterized protein | -0,19+/-0,06 |
| K00972 | 0,0050819 | UDP-N-acetylglucosamine/UDP-N-acetylgalactosamine diphosphorylase [EC:2.7.7.23 2.7.7.83] | -0,64+/-0,18 |
| K00162 | 0,0051201 | pyruvate dehydrogenase E1 component subunit beta [EC:1.2.4.1] | 0,91+/-0,26 |
| K01693 | 0,0051543 | imidazoleglycerol-phosphate dehydratase [EC:4.2.1.19] | -0,23+/-0,07 |
| K00979 | 0,0051543 | 3-deoxy-manno-octulosonate cytidylyltransferase (CMP-KDO synthetase) [EC:2.7.7.38] | 0,36+/-0,1 |
| K03545 | 0,0051647 | trigger factor | -0,23+/-0,07 |
| K09766 | 0,0052601 | uncharacterized protein | -0,63+/-0,18 |
| K02065 | 0,0052601 | phospholipid/cholesterol/gamma-HCH transport system ATP-binding protein | 0,37+/-0,11 |
| K09770 | 0,0052695 | uncharacterized protein | -0,63+/-0,18 |
| K18979 | 0,0053197 | epoxyqueuosine reductase [EC:1.17.99.6] | 0,63+/-0,18 |
| K00688 | 0,005331 | glycogen phosphorylase [EC:2.4.1.1] | -0,25+/-0,07 |
| K02066 | 0,0054518 | phospholipid/cholesterol/gamma-HCH transport system permease protein | 0,39+/-0,11 |
| K01246 | 0,0054518 | DNA-3-methyladenine glycosylase I [EC:3.2.2.20] | 0,32+/-0,09 |
| K03518 | 0,0054518 | aerobic carbon-monoxide dehydrogenase small subunit [EC:1.2.5.3] | -0,38+/-0,11 |
| K03272 | 0,005459 | D-beta-D-heptose 7-phosphate kinase / D-beta-D-heptose 1-phosphate adenosyltransferase [EC:2.7.1.167 2.7.7.70] | 0,86+/-0,25 |
| K02303 | 0,005459 | uroporphyrin-III C-methyltransferase [EC:2.1.1.107] | 1,97+/-0,57 |
| K04720 | 0,0054721 | threonine-phosphate decarboxylase [EC:4.1.1.81] | -0,3+/-0,09 |
| K02414 | 0,0054754 | flagellar hook-length control protein FliK | -0,71+/-0,21 |
| K01104 | 0,0055099 | protein-tyrosine phosphatase [EC:3.1.3.48] | -0,19+/-0,06 |
| K11741 | 0,0055236 | quaternary ammonium compound-resistance protein SugE | 0,42+/-0,12 |
| K00384 | 0,0055345 | thioredoxin reductase (NADPH) [EC:1.8.1.9] | -0,13+/-0,04 |
| K01912 | 0,0055657 | phenylacetate-CoA ligase [EC:6.2.1.30] | 0,31+/-0,09 |
| K17218 | 0,0056688 | sulfide:quinone oxidoreductase [EC:1.8.5.4] | 0,88+/-0,26 |
| K13821 | 0,0056692 | RHH-type transcriptional regulator, proline utilization regulon repressor / proline dehydrogenase / delta 1-pyrroline-5-carboxylate dehydrogenase [EC:1.5.5.2 1.2.1.88] | 1,56+/-0,45 |
| K03466 | 0,005704 | DNA segregation ATPase FtsK/SpoIIIE, S-DNA-T family | -0,13+/-0,04 |
| K03565 | 0,0057745 | regulatory protein | -0,17+/-0,05 |
| K00595 | 0,0057962 | precorrin-6B C5,15-methyltransferase / cobalt-precorrin-6B C5,C15-methyltransferase [EC:2.1.1.132 2.1.1.289 2.1.1.196] | -0,3+/-0,09 |
| K19225 | 0,0058518 | rhomboid protease GluP [EC:3.4.21.105] | -0,43+/-0,13 |
| K06906 | 0,0058658 | phage tail protein | 1,85+/-0,54 |
| K06374 | 0,005956 | spore maturation protein B | -0,46+/-0,13 |
| K03305 | 0,0059898 | proton-dependent oligopeptide transporter, POT family | 0,38+/-0,11 |
| K02073 | 0,0059898 | D-methionine transport system substrate-binding protein | -0,35+/-0,1 |
| K13626 | 0,0059937 | flagellar assembly factor FliW | -0,54+/-0,16 |
| K00610 | 0,0060269 | aspartate carbamoyltransferase regulatory subunit | -0,3+/-0,09 |
| K09940 | 0,0060319 | uncharacterized protein | 1,33+/-0,39 |
| K07040 | 0,0060824 | DUF177 domain-containing protein | -0,23+/-0,07 |
| K00859 | 0,0061241 | dephospho-CoA kinase [EC:2.7.1.24] | -0,16+/-0,05 |
| K09749 | 0,0061646 | uncharacterized protein | -0,52+/-0,15 |
| K03498 | 0,0061646 | trk/ktr system potassium uptake protein | -0,2+/-0,06 |
| K01179 | 0,006237 | endoglucanase [EC:3.2.1.4] | 0,59+/-0,17 |
| K03781 | 0,0063159 | catalase [EC:1.11.1.6] | 0,62+/-0,18 |
| K03770 | 0,0063968 | peptidyl-prolyl cis-trans isomerase D [EC:5.2.1.8] | 0,38+/-0,11 |
| K00381 | 0,0064239 | sulfite reductase (NADPH) hemoprotein beta-component [EC:1.8.1.2] | 1,63+/-0,48 |
| K07777 | 0,0065063 | two-component system, NarL family, sensor histidine kinase DegS [EC:2.7.13.3] | -0,64+/-0,19 |
| K07813 | 0,0065063 | accessory gene regulator B | -0,43+/-0,13 |
| K00945 | 0,0065749 | CMP/dCMP kinase [EC:2.7.4.25] | -0,19+/-0,06 |
| K07088 | 0,0066711 | uncharacterized | -0,25+/-0,07 |
| K00395 | 0,006706 | adenylylsulfate reductase, subunit B [EC:1.8.99.2] | -0,53+/-0,16 |
| K17318 | 0,0067114 | putative aldouronate transport system substrate-binding protein | -0,42+/-0,12 |
| K06041 | 0,006724 | arabinose-5-phosphate isomerase [EC:5.3.1.13] | 0,35+/-0,11 |
| K04041 | 0,0067593 | fructose-1,6-bisphosphatase III [EC:3.1.3.11] | -0,29+/-0,09 |
| K07315 | 0,0067644 | phosphoserine phosphatase RsbU/P [EC:3.1.3.3] | 0,85+/-0,25 |
| K03426 | 0,0068196 | NAD+ diphosphatase [EC:3.6.1.22] | -0,28+/-0,08 |
| K06941 | 0,0068196 | 23S rRNA (adenine2503-C2)-methyltransferase [EC:2.1.1.192] | -0,15+/-0,04 |
| K01992 | 0,0068512 | ABC-2 type transport system permease protein | -0,14+/-0,04 |
| K06183 | 0,0069113 | 16S rRNA pseudouridine516 synthase [EC:5.4.99.19] | -0,24+/-0,07 |
| K13280 | 0,0069429 | signal peptidase I [EC:3.4.21.89] | -0,46+/-0,14 |
| K01534 | 0,0069753 | Zn2+/Cd2+-exporting ATPase [EC:7.2.2.12 7.2.2.21] | -0,2+/-0,06 |
| K04019 | 0,0069843 | ethanolamine utilization protein EutA | 1,14+/-0,34 |
| K07736 | 0,0070807 | CarD family transcriptional regulator, regulator of rRNA transcription | -0,36+/-0,11 |
| K00611 | 0,0070897 | ornithine carbamoyltransferase [EC:2.1.3.3] | -0,28+/-0,08 |
| K13497 | 0,0071093 | anthranilate synthase/phosphoribosyltransferase [EC:4.1.3.27 2.4.2.18] | 1,2+/-0,36 |
| K18214 | 0,0071282 | MFS transporter, DHA3 family, tetracycline resistance protein | 1,54+/-0,46 |
| K02626 | 0,0071282 | arginine decarboxylase [EC:4.1.1.19] | 0,88+/-0,26 |
| K17076 | 0,0071439 | putative lysine transport system ATP-binding protein | -0,54+/-0,16 |
| K02575 | 0,0071439 | MFS transporter, NNP family, nitrate/nitrite transporter | 1,2+/-0,36 |
| K18349 | 0,0071597 | two-component system, OmpR family, response regulator VanR | -0,46+/-0,14 |
| K04068 | 0,0071597 | anaerobic ribonucleoside-triphosphate reductase activating protein [EC:1.97.1.4] | -0,17+/-0,05 |
| K13607 | 0,0071945 | cinnamoyl-CoA:phenyllactate CoA-transferase [EC:2.8.3.17] | 1,67+/-0,5 |
| K03569 | 0,0071945 | rod shape-determining protein MreB and related proteins | -0,18+/-0,05 |
| K07127 | 0,0072818 | 5-hydroxyisourate hydrolase [EC:3.5.2.17] | 2,09+/-0,63 |
| K04758 | 0,0072944 | ferrous iron transport protein A | -0,25+/-0,07 |
| K14161 | 0,0073037 | protein ImuB | 2,18+/-0,65 |
| K07400 | 0,0073184 | Fe/S biogenesis protein NfuA | 1,29+/-0,39 |
| K01734 | 0,0073547 | methylglyoxal synthase [EC:4.2.3.3] | -0,25+/-0,08 |
| K03501 | 0,0073983 | 16S rRNA (guanine527-N7)-methyltransferase [EC:2.1.1.170] | -0,15+/-0,05 |
| K07032 | 0,0074606 | uncharacterized protein | 1,39+/-0,42 |
| K16881 | 0,0075253 | mannose-1-phosphate guanylyltransferase / phosphomannomutase [EC:2.7.7.13 5.4.2.8] | 1,3+/-0,39 |
| K00912 | 0,0075254 | tetraacyldisaccharide 4'-kinase [EC:2.7.1.130] | 0,35+/-0,1 |
| K12340 | 0,0075254 | outer membrane protein | 0,38+/-0,11 |
| K00108 | 0,0076626 | choline dehydrogenase [EC:1.1.99.1] | 2,19+/-0,66 |
| K07729 | 0,0077437 | putative transcriptional regulator | -0,31+/-0,09 |
| K06217 | 0,0078023 | phosphate starvation-inducible protein PhoH and related proteins | -0,15+/-0,04 |
| K00784 | 0,0078023 | ribonuclease Z [EC:3.1.26.11] | -0,19+/-0,06 |
| K01939 | 0,0078295 | adenylosuccinate synthase [EC:6.3.4.4] | -0,16+/-0,05 |
| K03092 | 0,0079125 | RNA polymerase sigma-54 factor | 0,29+/-0,09 |
| K03924 | 0,0079147 | MoxR-like ATPase [EC:3.6.3.-] | 0,27+/-0,08 |
| K03782 | 0,007938 | catalase-peroxidase [EC:1.11.1.21] | 1,91+/-0,58 |
| K07397 | 0,007938 | putative redox protein | 0,77+/-0,23 |
| K06965 | 0,007938 | protein pelota | 1,66+/-0,5 |
| K15016 | 0,007938 | enoyl-CoA hydratase / 3-hydroxyacyl-CoA dehydrogenase [EC:4.2.1.17 1.1.1.35] | 1,66+/-0,5 |
| K18123 | 0,007938 | 4-hydroxy-2-oxoglutarate aldolase [EC:4.1.3.16] | 1,66+/-0,5 |
| K18313 | 0,007938 | succinyl-CoA---D-citramalate CoA-transferase [EC:2.8.3.20] | 1,66+/-0,5 |
| K02078 | 0,0080954 | acyl carrier protein | -0,15+/-0,04 |
| K07093 | 0,0081126 | uncharacterized protein | 2+/-0,61 |
| K15986 | 0,0081274 | manganese-dependent inorganic pyrophosphatase [EC:3.6.1.1] | -0,27+/-0,08 |
| K00620 | 0,0083021 | glutamate N-acetyltransferase / amino-acid N-acetyltransferase [EC:2.3.1.35 2.3.1.1] | -0,25+/-0,08 |
| K03469 | 0,0083021 | ribonuclease HI [EC:3.1.26.4] | -0,14+/-0,04 |
| K02291 | 0,0083021 | 15-cis-phytoene synthase [EC:2.5.1.32] | 1,67+/-0,51 |
| K03449 | 0,0083021 | MFS transporter, CP family, cyanate transporter | 1,38+/-0,42 |
| K09825 | 0,0083204 | Fur family transcriptional regulator, peroxide stress response regulator | -0,27+/-0,08 |
| K00761 | 0,0084974 | uracil phosphoribosyltransferase [EC:2.4.2.9] | -0,15+/-0,04 |
| K00950 | 0,008508 | 2-amino-4-hydroxy-6-hydroxymethyldihydropteridine diphosphokinase [EC:2.7.6.3] | 0,3+/-0,09 |
| K00948 | 0,008508 | ribose-phosphate pyrophosphokinase [EC:2.7.6.1] | -0,12+/-0,04 |
| K02453 | 0,0085113 | general secretion pathway protein D | 1,63+/-0,5 |
| K07787 | 0,0085654 | copper/silver efflux system protein | 0,81+/-0,25 |
| K07798 | 0,0085654 | membrane fusion protein, copper/silver efflux system | 0,81+/-0,25 |
| K00014 | 0,0085654 | shikimate dehydrogenase [EC:1.1.1.25] | -0,13+/-0,04 |
| K00135 | 0,0086533 | succinate-semialdehyde dehydrogenase / glutarate-semialdehyde dehydrogenase [EC:1.2.1.16 1.2.1.79 1.2.1.20] | 1,24+/-0,38 |
| K02860 | 0,0087131 | 16S rRNA processing protein RimM | -0,15+/-0,05 |
| K00651 | 0,0087209 | homoserine O-succinyltransferase/O-acetyltransferase [EC:2.3.1.46 2.3.1.31] | -0,25+/-0,08 |
| K00104 | 0,0087209 | glycolate dehydrogenase FAD-linked subunit [EC:1.1.99.14] | 0,67+/-0,21 |
| K01637 | 0,0087305 | isocitrate lyase [EC:4.1.3.1] | 2,02+/-0,62 |
| K03183 | 0,0087377 | demethylmenaquinone methyltransferase / 2-methoxy-6-polyprenyl-1,4-benzoquinol methylase [EC:2.1.1.163 2.1.1.201] | 0,31+/-0,1 |
| K12952 | 0,0087693 | cation-transporting P-type ATPase E [EC:7.2.2.-] | -0,41+/-0,13 |
| K02301 | 0,0087693 | uncharacterized | 1,48+/-0,45 |
| K06077 | 0,0087693 | outer membrane lipoprotein SlyB | 1,39+/-0,43 |
| K01971 | 0,0087699 | bifunctional non-homologous end joining protein LigD [EC:6.5.1.1] | 1,12+/-0,35 |
| K11071 | 0,0088331 | spermidine/putrescine transport system permease protein | -0,24+/-0,07 |
| K01895 | 0,0088346 | acetyl-CoA synthetase [EC:6.2.1.1] | 0,38+/-0,12 |
| K18139 | 0,0088346 | outer membrane protein, multidrug efflux system | 0,44+/-0,13 |
| K01687 | 0,008894 | dihydroxy-acid dehydratase [EC:4.2.1.9] | -0,13+/-0,04 |
| K03743 | 0,0089276 | nicotinamide-nucleotide amidase [EC:3.5.1.42] | -0,49+/-0,15 |
| K05782 | 0,0089276 | benzoate membrane transport protein | 1,68+/-0,52 |
| K14162 | 0,0089734 | error-prone DNA polymerase [EC:2.7.7.7] | 2,12+/-0,65 |
| K11070 | 0,0089734 | spermidine/putrescine transport system permease protein | -0,24+/-0,07 |
| K00303 | 0,0090625 | sarcosine oxidase, subunit beta [EC:1.5.3.24 1.5.3.1] | 1,52+/-0,47 |
| K01035 | 0,0090625 | acetate CoA/acetoacetate CoA-transferase beta subunit [EC:2.8.3.8 2.8.3.9] | 0,74+/-0,23 |
| K06995 | 0,0090718 | uncharacterized protein | 1,89+/-0,58 |
| K00954 | 0,0091343 | pantetheine-phosphate adenylyltransferase [EC:2.7.7.3] | -0,14+/-0,04 |
| K19350 | 0,0093714 | lincosamide and streptogramin A transport system ATP-binding/permease protein | -0,47+/-0,15 |
| K19422 | 0,009382 | glycosyltransferase EpsD [EC:2.4.-.-] | 1,48+/-0,46 |
| K19118 | 0,009382 | CRISPR-associated protein Csd2 | 0,72+/-0,22 |
| K05970 | 0,009382 | sialate O-acetylesterase [EC:3.1.1.53] | 0,35+/-0,11 |
| K02907 | 0,0093873 | large subunit ribosomal protein L30 | -0,16+/-0,05 |
| K02046 | 0,0093873 | sulfate/thiosulfate transport system permease protein | -0,48+/-0,15 |
| K02047 | 0,0093873 | sulfate/thiosulfate transport system permease protein | -0,48+/-0,15 |
| K18350 | 0,0094543 | two-component system, OmpR family, sensor histidine kinase VanS [EC:2.7.13.3] | -0,46+/-0,14 |
| K06410 | 0,0095025 | dipicolinate synthase subunit A | -0,4+/-0,12 |
| K02033 | 0,009527 | peptide/nickel transport system permease protein | -0,22+/-0,07 |
| K00231 | 0,0095498 | protoporphyrinogen/coproporphyrinogen III oxidase [EC:1.3.3.4 1.3.3.15] | 0,68+/-0,21 |
| K04485 | 0,0096419 | DNA repair protein RadA/Sms | -0,16+/-0,05 |
| K01034 | 0,0096475 | acetate CoA/acetoacetate CoA-transferase alpha subunit [EC:2.8.3.8 2.8.3.9] | 0,73+/-0,23 |
| K03169 | 0,0097876 | DNA topoisomerase III [EC:5.6.2.1] | -0,21+/-0,07 |
| K02045 | 0,0097876 | sulfate/thiosulfate transport system ATP-binding protein [EC:7.3.2.3] | -0,47+/-0,15 |
| K00394 | 0,0097977 | adenylylsulfate reductase, subunit A [EC:1.8.99.2] | -0,51+/-0,16 |
| K03644 | 0,0098211 | lipoyl synthase [EC:2.8.1.8] | 0,41+/-0,13 |
| K09015 | 0,0098211 | Fe-S cluster assembly protein SufD | 0,33+/-0,1 |
| K01924 | 0,0098905 | UDP-N-acetylmuramate--alanine ligase [EC:6.3.2.8] | -0,14+/-0,04 |
| K00097 | 0,0099895 | 4-hydroxythreonine-4-phosphate dehydrogenase [EC:1.1.1.262] | 0,28+/-0,09 |
| K02411 | 0,010107 | flagellar assembly protein FliH | -0,47+/-0,15 |
| K02536 | 0,010107 | UDP-3-O-[3-hydroxymyristoyl] glucosamine N-acyltransferase [EC:2.3.1.191] | 0,33+/-0,1 |
| K02825 | 0,010238 | pyrimidine operon attenuation protein / uracil phosphoribosyltransferase [EC:2.4.2.9] | 0,71+/-0,22 |
| K07165 | 0,010391 | transmembrane sensor | 2,92+/-0,92 |
| K04084 | 0,010413 | thioredoxin:protein disulfide reductase [EC:1.8.4.16] | 0,35+/-0,11 |
| K10806 | 0,010413 | acyl-CoA thioesterase YciA [EC:3.1.2.-] | 1,5+/-0,47 |
| K06213 | 0,010472 | magnesium transporter | -0,28+/-0,09 |
| K17074 | 0,010472 | putative lysine transport system permease protein | -0,52+/-0,16 |
| K02232 | 0,010472 | adenosylcobyric acid synthase [EC:6.3.5.10] | -0,25+/-0,08 |
| K19405 | 0,010498 | protein arginine kinase [EC:2.7.14.1] | 1,07+/-0,34 |
| K07140 | 0,010498 | uncharacterized protein | 1,99+/-0,62 |
| K01081 | 0,010498 | 5'-nucleotidase [EC:3.1.3.5] | 0,33+/-0,1 |
| K03499 | 0,010498 | trk/ktr system potassium uptake protein | -0,2+/-0,06 |
| K07250 | 0,010498 | 4-aminobutyrate aminotransferase / (S)-3-amino-2-methylpropionate transaminase / 5-aminovalerate transaminase [EC:2.6.1.19 2.6.1.22 2.6.1.48] | 2,25+/-0,71 |
| K01434 | 0,010498 | penicillin G amidase [EC:3.5.1.11] | 1,95+/-0,61 |
| K02398 | 0,010498 | negative regulator of flagellin synthesis FlgM | -0,47+/-0,15 |
| K00949 | 0,010498 | thiamine pyrophosphokinase [EC:2.7.6.2] | -0,26+/-0,08 |
| K17865 | 0,010498 | 3-hydroxybutyryl-CoA dehydratase [EC:4.2.1.55] | 1,17+/-0,37 |
| K00254 | 0,010519 | dihydroorotate dehydrogenase [EC:1.3.5.2] | 0,54+/-0,17 |
| K02401 | 0,010566 | flagellar biosynthesis protein FlhB | -0,44+/-0,14 |
| K05601 | 0,010566 | hydroxylamine reductase [EC:1.7.99.1] | -0,3+/-0,09 |
| K07091 | 0,010619 | lipopolysaccharide export system permease protein | 0,35+/-0,11 |
| K19427 | 0,010637 | glycosyltransferase EpsJ [EC:2.4.-.-] | 1,53+/-0,48 |
| K01515 | 0,010637 | ADP-ribose diphosphatase [EC:3.6.1.13 3.6.1.-] | -0,21+/-0,07 |
| K01669 | 0,010651 | deoxyribodipyrimidine photo-lyase [EC:4.1.99.3] | 1,63+/-0,51 |
| K01627 | 0,010655 | 2-dehydro-3-deoxyphosphooctonate aldolase (KDO 8-P synthase) [EC:2.5.1.55] | 0,34+/-0,11 |
| K02837 | 0,010674 | peptide chain release factor 3 | -0,16+/-0,05 |
| K02417 | 0,010674 | flagellar motor switch protein FliN | -0,44+/-0,14 |
| K02421 | 0,010674 | flagellar biosynthesis protein FliR | -0,44+/-0,14 |
| K08678 | 0,010686 | UDP-glucuronate decarboxylase [EC:4.1.1.35] | 0,56+/-0,18 |
| K09781 | 0,010836 | uncharacterized protein | 2,09+/-0,66 |
| K02418 | 0,010854 | flagellar protein FliO/FliZ | -0,47+/-0,15 |
| K02226 | 0,010907 | alpha-ribazole phosphatase [EC:3.1.3.73] | -0,27+/-0,08 |
| K02409 | 0,010992 | flagellar M-ring protein FliF | -0,46+/-0,15 |
| K02389 | 0,010995 | flagellar basal-body rod modification protein FlgD | -0,46+/-0,14 |
| K05541 | 0,010995 | tRNA-dihydrouridine synthase C [EC:1.-.-.-] | 1,63+/-0,52 |
| K01638 | 0,010995 | malate synthase [EC:2.3.3.9] | 1,95+/-0,62 |
| K02416 | 0,01101 | flagellar motor switch protein FliM | -0,44+/-0,14 |
| K07248 | 0,011037 | lactaldehyde dehydrogenase / glycolaldehyde dehydrogenase [EC:1.2.1.22 1.2.1.21] | 0,82+/-0,26 |
| K13766 | 0,011037 | methylglutaconyl-CoA hydratase [EC:4.2.1.18] | 2,1+/-0,66 |
| K02410 | 0,011037 | flagellar motor switch protein FliG | -0,44+/-0,14 |
| K00748 | 0,011127 | lipid-A-disaccharide synthase [EC:2.4.1.182] | 0,33+/-0,11 |
| K02412 | 0,011141 | flagellum-specific ATP synthase [EC:7.4.2.8] | -0,44+/-0,14 |
| K02420 | 0,01118 | flagellar biosynthesis protein FliQ | -0,43+/-0,14 |
| K02408 | 0,01118 | flagellar hook-basal body complex protein FliE | -0,44+/-0,14 |
| K02387 | 0,01118 | flagellar basal-body rod protein FlgB | -0,44+/-0,14 |
| K07267 | 0,01118 | porin | 1,6+/-0,51 |
| K02400 | 0,01118 | flagellar biosynthesis protein FlhA | -0,44+/-0,14 |
| K07277 | 0,011216 | outer membrane protein insertion porin family | 0,33+/-0,11 |
| K02419 | 0,011252 | flagellar biosynthesis protein FliP | -0,43+/-0,14 |
| K02397 | 0,011421 | flagellar hook-associated protein 3 FlgL | -0,46+/-0,15 |
| K06861 | 0,011428 | lipopolysaccharide export system ATP-binding protein [EC:7.5.2.5] | 0,33+/-0,11 |
| K09808 | 0,011625 | lipoprotein-releasing system permease protein | 0,33+/-0,11 |
| K00558 | 0,011719 | DNA (cytosine-5)-methyltransferase 1 [EC:2.1.1.37] | -0,18+/-0,06 |
| K06877 | 0,011794 | DEAD/DEAH box helicase domain-containing protein | 1,43+/-0,46 |
| K02523 | 0,011794 | octaprenyl-diphosphate synthase [EC:2.5.1.90] | 0,35+/-0,11 |
| K08217 | 0,011799 | MFS transporter, DHA3 family, macrolide efflux protein | 0,47+/-0,15 |
| K02413 | 0,011799 | flagellar protein FliJ | -0,46+/-0,15 |
| K13993 | 0,011799 | HSP20 family protein | 0,29+/-0,09 |
| K03587 | 0,011799 | cell division protein FtsI (penicillin-binding protein 3) [EC:3.4.16.4] | 0,3+/-0,09 |
| K03595 | 0,011921 | GTPase | -0,15+/-0,05 |
| K03825 | 0,011987 | L-phenylalanine/L-methionine N-acetyltransferase [EC:2.3.1.53 2.3.1.-] | 1,1+/-0,35 |
| K15268 | 0,012068 | O-acetylserine/cysteine efflux transporter | 1,75+/-0,56 |
| K03488 | 0,012068 | beta-glucoside operon transcriptional antiterminator | -0,39+/-0,12 |
| K03579 | 0,012289 | ATP-dependent helicase HrpB [EC:3.6.4.13] | 1,24+/-0,4 |
| K05807 | 0,01229 | outer membrane protein assembly factor BamD | 0,35+/-0,11 |
| K00208 | 0,012486 | enoyl-[acyl-carrier protein] reductase I [EC:1.3.1.9 1.3.1.10] | 0,39+/-0,13 |
| K02429 | 0,012555 | MFS transporter, FHS family, L-fucose permease | 0,33+/-0,11 |
| K00332 | 0,012555 | NADH-quinone oxidoreductase subunit C [EC:7.1.1.2] | 1,59+/-0,51 |
| K00333 | 0,012555 | NADH-quinone oxidoreductase subunit D [EC:7.1.1.2] | 1,59+/-0,51 |
| K17733 | 0,012555 | peptidoglycan LD-endopeptidase CwlK [EC:3.4.-.-] | 1,45+/-0,47 |
| K02388 | 0,012683 | flagellar basal-body rod protein FlgC | -0,43+/-0,14 |
| K02274 | 0,012683 | cytochrome c oxidase subunit I [EC:7.1.1.9] | 1,36+/-0,44 |
| K01776 | 0,012712 | glutamate racemase [EC:5.1.1.3] | -0,15+/-0,05 |
| K01012 | 0,012712 | biotin synthase [EC:2.8.1.6] | -0,22+/-0,07 |
| K03455 | 0,012766 | K+:H+ antiporter | 0,34+/-0,11 |
| K00111 | 0,01278 | glycerol-3-phosphate dehydrogenase [EC:1.1.5.3] | -0,17+/-0,06 |
| K06411 | 0,012794 | dipicolinate synthase subunit B | -0,37+/-0,12 |
| K00625 | 0,012913 | phosphate acetyltransferase [EC:2.3.1.8] | -0,26+/-0,08 |
| K00426 | 0,012982 | cytochrome bd ubiquinol oxidase subunit II [EC:7.1.1.7] | 0,33+/-0,11 |
| K08591 | 0,013108 | acyl phosphate:glycerol-3-phosphate acyltransferase [EC:2.3.1.275] | -0,18+/-0,06 |
| K00185 | 0,013203 | dimethyl sulfoxide reductase membrane subunit | 1,3+/-0,42 |
| K06317 | 0,013332 | inhibitor of the pro-sigma K processing machinery | -0,47+/-0,15 |
| K03801 | 0,013352 | lipoyl(octanoyl) transferase [EC:2.3.1.181] | 0,4+/-0,13 |
| K00425 | 0,013409 | cytochrome bd ubiquinol oxidase subunit I [EC:7.1.1.7] | 0,33+/-0,11 |
| K01625 | 0,013515 | 2-dehydro-3-deoxyphosphogluconate aldolase / (4S)-4-hydroxy-2-oxoglutarate aldolase [EC:4.1.2.14 4.1.3.42] | -0,17+/-0,06 |
| K10544 | 0,013573 | D-xylose transport system permease protein | 2+/-0,65 |
| K01676 | 0,013665 | fumarate hydratase, class I [EC:4.2.1.2] | 0,34+/-0,11 |
| K00812 | 0,013706 | aspartate aminotransferase [EC:2.6.1.1] | 0,36+/-0,12 |
| K07033 | 0,013777 | uncharacterized protein | -0,43+/-0,14 |
| K04030 | 0,013797 | ethanolamine utilization protein EutQ | 1,04+/-0,34 |
| K02259 | 0,013798 | heme a synthase [EC:1.17.99.9] | 1,37+/-0,45 |
| K07157 | 0,013877 | uncharacterized protein | 1,91+/-0,62 |
| K00864 | 0,0139 | glycerol kinase [EC:2.7.1.30] | -0,18+/-0,06 |
| K02549 | 0,014007 | o-succinylbenzoate synthase [EC:4.2.1.113] | 1,22+/-0,4 |
| K00831 | 0,014015 | phosphoserine aminotransferase [EC:2.6.1.52] | -0,15+/-0,05 |
| K02552 | 0,014015 | menaquinone-specific isochorismate synthase [EC:5.4.4.2] | 1,22+/-0,4 |
| K02276 | 0,01403 | cytochrome c oxidase subunit III [EC:7.1.1.9] | 1,32+/-0,43 |
| K01872 | 0,014075 | alanyl-tRNA synthetase [EC:6.1.1.7] | -0,16+/-0,05 |
| K08600 | 0,01434 | sortase B [EC:3.4.22.71] | -0,36+/-0,12 |
| K06603 | 0,014431 | flagellar protein FlaG | -0,51+/-0,17 |
| K18640 | 0,014623 | plasmid segregation protein ParM | -0,4+/-0,13 |
| K00019 | 0,014623 | 3-hydroxybutyrate dehydrogenase [EC:1.1.1.30] | 1,24+/-0,41 |
| K03610 | 0,014683 | septum site-determining protein MinC | -0,39+/-0,13 |
| K03086 | 0,0149 | RNA polymerase primary sigma factor | -0,22+/-0,07 |
| K00528 | 0,014901 | ferredoxin/flavodoxin---NADP+ reductase [EC:1.18.1.2 1.19.1.1] | -0,18+/-0,06 |
| K06871 | 0,014939 | uncharacterized protein | -0,2+/-0,07 |
| K13920 | 0,015191 | propanediol dehydratase small subunit [EC:4.2.1.28] | 1,2+/-0,4 |
| K00451 | 0,015191 | homogentisate 1,2-dioxygenase [EC:1.13.11.5] | 1,96+/-0,65 |
| K01969 | 0,015191 | 3-methylcrotonyl-CoA carboxylase beta subunit [EC:6.4.1.4] | 2,1+/-0,69 |
| K01733 | 0,01543 | threonine synthase [EC:4.2.3.1] | -0,13+/-0,04 |
| K00184 | 0,01543 | dimethyl sulfoxide reductase iron-sulfur subunit | 1,16+/-0,38 |
| K00786 | 0,015495 | beta-1,6-galactosyltransferase [EC:2.4.1.-] | -0,61+/-0,2 |
| K14267 | 0,015597 | N-succinyldiaminopimelate aminotransferase [EC:2.6.1.17] | 2,12+/-0,7 |
| K02454 | 0,015609 | general secretion pathway protein E [EC:7.4.2.8] | 1,02+/-0,34 |
| K01787 | 0,015878 | N-acylglucosamine 2-epimerase [EC:5.1.3.8] | 0,35+/-0,12 |
| K08311 | 0,01594 | putative (di)nucleoside polyphosphate hydrolase [EC:3.6.1.-] | 1,46+/-0,48 |
| K02116 | 0,015997 | ATP synthase protein I | 1,08+/-0,36 |
| K01703 | 0,016111 | 3-isopropylmalate/(R)-2-methylmalate dehydratase large subunit [EC:4.2.1.33 4.2.1.35] | -0,13+/-0,04 |
| K02621 | 0,016171 | topoisomerase IV subunit A [EC:5.6.2.2] | 0,27+/-0,09 |
| K07137 | 0,016176 | uncharacterized protein | -0,17+/-0,06 |
| K08641 | 0,016296 | zinc D-Ala-D-Ala dipeptidase [EC:3.4.13.22] | 0,37+/-0,12 |
| K00615 | 0,016448 | transketolase [EC:2.2.1.1] | -0,19+/-0,06 |
| K02622 | 0,016626 | topoisomerase IV subunit B [EC:5.6.2.2] | 0,27+/-0,09 |
| K11720 | 0,016806 | lipopolysaccharide export system permease protein | 0,34+/-0,11 |
| K03704 | 0,016849 | cold shock protein | -0,24+/-0,08 |
| K11903 | 0,016961 | type VI secretion system secreted protein Hcp | 1,71+/-0,57 |
| K15581 | 0,017172 | oligopeptide transport system permease protein | -0,29+/-0,1 |
| K07483 | 0,017275 | transposase | -0,37+/-0,12 |
| K00573 | 0,017304 | protein-L-isoaspartate(D-aspartate) O-methyltransferase [EC:2.1.1.77] | 1,63+/-0,55 |
| K01835 | 0,017312 | phosphoglucomutase [EC:5.4.2.2] | -0,16+/-0,05 |
| K14347 | 0,017382 | solute carrier family 10 (sodium/bile acid cotransporter), member 7 | 1,6+/-0,54 |
| K03386 | 0,017444 | peroxiredoxin 2/4 [EC:1.11.1.24] | 0,31+/-0,1 |
| K02049 | 0,017622 | NitT/TauT family transport system ATP-binding protein | -0,14+/-0,05 |
| K00971 | 0,017663 | mannose-1-phosphate guanylyltransferase [EC:2.7.7.13] | 0,32+/-0,11 |
| K00281 | 0,017663 | glycine cleavage system P protein (glycine dehydrogenase) [EC:1.4.4.2] | 0,57+/-0,19 |
| K10439 | 0,017712 | ribose transport system substrate-binding protein | -0,35+/-0,12 |
| K04771 | 0,017892 | serine protease Do [EC:3.4.21.107] | -0,2+/-0,07 |
| K01647 | 0,01808 | citrate synthase [EC:2.3.3.1] | -0,21+/-0,07 |
| K10947 | 0,018301 | PadR family transcriptional regulator | -0,2+/-0,07 |
| K03415 | 0,018301 | two-component system, chemotaxis family, chemotaxis protein CheV | -0,42+/-0,14 |
| K02404 | 0,018364 | flagellar biosynthesis protein FlhF | -0,49+/-0,17 |
| K03146 | 0,018364 | cysteine-dependent adenosine diphosphate thiazole synthase [EC:2.4.2.60] | 0,79+/-0,27 |
| K19271 | 0,018436 | chloramphenicol O-acetyltransferase type A [EC:2.3.1.28] | 0,34+/-0,12 |
| K06442 | 0,018588 | 23S rRNA (cytidine1920-2'-O)/16S rRNA (cytidine1409-2'-O)-methyltransferase [EC:2.1.1.226 2.1.1.227] | -0,22+/-0,07 |
| K13894 | 0,018994 | microcin C transport system permease protein | 1,78+/-0,61 |
| K13895 | 0,019138 | microcin C transport system permease protein | 1,78+/-0,6 |
| K00926 | 0,0192 | carbamate kinase [EC:2.7.2.2] | -0,32+/-0,11 |
| K02358 | 0,019275 | elongation factor Tu | -0,12+/-0,04 |
| K07396 | 0,019309 | putative protein-disulfide isomerase | 1,87+/-0,64 |
| K03772 | 0,019511 | FKBP-type peptidyl-prolyl cis-trans isomerase FkpA [EC:5.2.1.8] | 0,68+/-0,23 |
| K09812 | 0,019591 | cell division transport system ATP-binding protein | -0,15+/-0,05 |
| K01704 | 0,019739 | 3-isopropylmalate/(R)-2-methylmalate dehydratase small subunit [EC:4.2.1.33 4.2.1.35] | -0,12+/-0,04 |
| K00380 | 0,019795 | sulfite reductase (NADPH) flavoprotein alpha-component [EC:1.8.1.2] | 1,56+/-0,53 |
| K00163 | 0,01981 | pyruvate dehydrogenase E1 component [EC:1.2.4.1] | 1,68+/-0,57 |
| K00075 | 0,019862 | UDP-N-acetylmuramate dehydrogenase [EC:1.3.1.98] | -0,14+/-0,05 |
| K02342 | 0,019862 | DNA polymerase III subunit epsilon [EC:2.7.7.7] | 0,24+/-0,08 |
| K01438 | 0,019862 | acetylornithine deacetylase [EC:3.5.1.16] | 0,34+/-0,12 |
| K01555 | 0,019896 | fumarylacetoacetase [EC:3.7.1.2] | 2,06+/-0,7 |
| K09765 | 0,019896 | epoxyqueuosine reductase [EC:1.17.99.6] | -0,21+/-0,07 |
| K18829 | 0,020008 | antitoxin VapB | 1,49+/-0,51 |
| K00344 | 0,020008 | NADPH:quinone reductase [EC:1.6.5.5] | 1,13+/-0,39 |
| K00879 | 0,020051 | L-fuculokinase [EC:2.7.1.51] | 1,5+/-0,51 |
| K08738 | 0,020051 | cytochrome c | 1,68+/-0,58 |
| K03978 | 0,020073 | GTP-binding protein | -0,14+/-0,05 |
| K01933 | 0,020154 | phosphoribosylformylglycinamidine cyclo-ligase [EC:6.3.3.1] | -0,11+/-0,04 |
| K07713 | 0,020238 | two-component system, NtrC family, response regulator HydG | 0,58+/-0,2 |
| K01682 | 0,020238 | aconitate hydratase 2 / 2-methylisocitrate dehydratase [EC:4.2.1.3 4.2.1.99] | 2,65+/-0,91 |
| K01754 | 0,020284 | threonine dehydratase [EC:4.3.1.19] | -0,17+/-0,06 |
| K03802 | 0,020334 | cyanophycin synthetase [EC:6.3.2.29 6.3.2.30] | 1,72+/-0,59 |
| K07402 | 0,020488 | xanthine dehydrogenase accessory factor | -0,29+/-0,1 |
| K08680 | 0,021001 | 2-succinyl-6-hydroxy-2,4-cyclohexadiene-1-carboxylate synthase [EC:4.2.99.20] | 1,23+/-0,42 |
| K03411 | 0,021089 | chemotaxis protein CheD [EC:3.5.1.44] | -0,43+/-0,15 |
| K00239 | 0,02141 | succinate dehydrogenase flavoprotein subunit [EC:1.3.5.1] | 0,26+/-0,09 |
| K09701 | 0,02141 | uncharacterized protein | 2,04+/-0,71 |
| K03593 | 0,02141 | ATP-binding protein involved in chromosome partitioning | 0,3+/-0,1 |
| K01092 | 0,021417 | myo-inositol-1(or 4)-monophosphatase [EC:3.1.3.25] | 0,23+/-0,08 |
| K00941 | 0,021418 | hydroxymethylpyrimidine/phosphomethylpyrimidine kinase [EC:2.7.1.49 2.7.4.7] | -0,17+/-0,06 |
| K03182 | 0,021498 | 4-hydroxy-3-polyprenylbenzoate decarboxylase [EC:4.1.1.98] | 0,5+/-0,17 |
| K00925 | 0,021498 | acetate kinase [EC:2.7.2.1] | -0,15+/-0,05 |
| K00003 | 0,021498 | homoserine dehydrogenase [EC:1.1.1.3] | -0,15+/-0,05 |
| K03296 | 0,021511 | hydrophobic/amphiphilic exporter-1 (mainly G- bacteria), HAE1 family | 0,2+/-0,07 |
| K17828 | 0,021584 | dihydroorotate dehydrogenase (NAD+) catalytic subunit [EC:1.3.1.14] | -0,14+/-0,05 |
| K01684 | 0,021748 | galactonate dehydratase [EC:4.2.1.6] | 0,56+/-0,19 |
| K01874 | 0,021885 | methionyl-tRNA synthetase [EC:6.1.1.10] | -0,11+/-0,04 |
| K09793 | 0,021915 | uncharacterized protein | 0,55+/-0,19 |
| K03186 | 0,021965 | flavin prenyltransferase [EC:2.5.1.129] | 0,49+/-0,17 |
| K03588 | 0,021993 | cell division protein FtsW | -0,14+/-0,05 |
| K00001 | 0,022048 | alcohol dehydrogenase [EC:1.1.1.1] | 0,36+/-0,12 |
| K13919 | 0,022065 | propanediol dehydratase medium subunit [EC:4.2.1.28] | 1,14+/-0,4 |
| K03473 | 0,022165 | erythronate-4-phosphate dehydrogenase [EC:1.1.1.290] | 0,39+/-0,13 |
| K05772 | 0,022175 | tungstate transport system substrate-binding protein | 1,29+/-0,45 |
| K06872 | 0,022209 | uncharacterized protein | -0,19+/-0,07 |
| K00176 | 0,022237 | 2-oxoglutarate ferredoxin oxidoreductase subunit delta [EC:1.2.7.3] | 0,35+/-0,12 |
| K04562 | 0,022467 | flagellar biosynthesis protein FlhG | -0,48+/-0,17 |
| K07576 | 0,022531 | metallo-beta-lactamase family protein | -0,23+/-0,08 |
| K14682 | 0,022665 | amino-acid N-acetyltransferase [EC:2.3.1.1] | 1,72+/-0,6 |
| K07217 | 0,022695 | manganese catalase [EC:1.11.1.6] | 0,65+/-0,23 |
| K02405 | 0,022732 | RNA polymerase sigma factor FliA | -0,43+/-0,15 |
| K01447 | 0,022732 | N-acetylmuramoyl-L-alanine amidase [EC:3.5.1.28] | 0,53+/-0,18 |
| K02548 | 0,022876 | 1,4-dihydroxy-2-naphthoate polyprenyltransferase [EC:2.5.1.74] | 0,29+/-0,1 |
| K01414 | 0,02304 | oligopeptidase A [EC:3.4.24.70] | 0,78+/-0,27 |
| K01489 | 0,02304 | cytidine deaminase [EC:3.5.4.5] | -0,16+/-0,06 |
| K03547 | 0,02304 | DNA repair protein SbcD/Mre11 | -0,2+/-0,07 |
| K02231 | 0,023348 | adenosylcobinamide kinase / adenosylcobinamide-phosphate guanylyltransferase [EC:2.7.1.156 2.7.7.62] | -0,22+/-0,08 |
| K01699 | 0,023348 | propanediol dehydratase large subunit [EC:4.2.1.28] | 1,2+/-0,42 |
| K00883 | 0,023601 | 2-dehydro-3-deoxygalactonokinase [EC:2.7.1.58] | 1,7+/-0,59 |
| K01586 | 0,023615 | diaminopimelate decarboxylase [EC:4.1.1.20] | -0,11+/-0,04 |
| K05773 | 0,023627 | tungstate transport system permease protein | 1,28+/-0,45 |
| K03311 | 0,023627 | branched-chain amino acid:cation transporter, LIVCS family | -0,32+/-0,11 |
| K12410 | 0,023788 | NAD-dependent protein deacetylase/lipoamidase [EC:2.3.1.286 2.3.1.313] | -0,14+/-0,05 |
| K00457 | 0,02401 | 4-hydroxyphenylpyruvate dioxygenase [EC:1.13.11.27] | 1,5+/-0,53 |
| K01118 | 0,024123 | FMN-dependent NADH-azoreductase [EC:1.7.1.17] | 0,57+/-0,2 |
| K00048 | 0,024213 | lactaldehyde reductase [EC:1.1.1.77] | -0,22+/-0,08 |
| K05919 | 0,02426 | superoxide reductase [EC:1.15.1.2] | -0,3+/-0,1 |
| K03615 | 0,024364 | H+/Na+-translocating ferredoxin:NAD+ oxidoreductase subunit C [EC:7.1.1.11 7.2.1.2] | -0,16+/-0,05 |
| K02390 | 0,024421 | flagellar hook protein FlgE | -0,39+/-0,14 |
| K19411 | 0,024475 | protein arginine kinase activator | 0,93+/-0,33 |
| K17320 | 0,024511 | putative aldouronate transport system permease protein | -0,38+/-0,13 |
| K03655 | 0,024542 | ATP-dependent DNA helicase RecG [EC:5.6.2.4] | -0,14+/-0,05 |
| K01420 | 0,024542 | CRP/FNR family transcriptional regulator, anaerobic regulatory protein | -0,26+/-0,09 |
| K01679 | 0,024957 | fumarate hydratase, class II [EC:4.2.1.2] | 0,57+/-0,2 |
| K02004 | 0,025058 | putative ABC transport system permease protein | -0,16+/-0,06 |
| K00963 | 0,025083 | UTP--glucose-1-phosphate uridylyltransferase [EC:2.7.7.9] | -0,21+/-0,07 |
| K07148 | 0,025083 | uncharacterized protein | 0,55+/-0,19 |
| K03524 | 0,025194 | BirA family transcriptional regulator, biotin operon repressor / biotin---[acetyl-CoA-carboxylase] ligase [EC:6.3.4.15] | -0,14+/-0,05 |
| K07082 | 0,025194 | UPF0755 protein | -0,14+/-0,05 |
| K07322 | 0,025194 | regulator of cell morphogenesis and NO signaling | 0,31+/-0,11 |
| K00029 | 0,025218 | malate dehydrogenase (oxaloacetate-decarboxylating)(NADP+) [EC:1.1.1.40] | 0,31+/-0,11 |
| K01596 | 0,025292 | phosphoenolpyruvate carboxykinase (GTP) [EC:4.1.1.32] | 0,59+/-0,21 |
| K00567 | 0,025347 | methylated-DNA-[protein]-cysteine S-methyltransferase [EC:2.1.1.63] | 0,15+/-0,05 |
| K11904 | 0,025536 | type VI secretion system secreted protein VgrG | 1,58+/-0,56 |
| K00924 | 0,025614 | kinase [EC:2.7.1.-] | 1,72+/-0,61 |
| K03437 | 0,025926 | RNA methyltransferase, TrmH family | -0,15+/-0,05 |
| K07095 | 0,025934 | uncharacterized protein | -0,18+/-0,06 |
| K02015 | 0,025934 | iron complex transport system permease protein | -0,11+/-0,04 |
| K01284 | 0,025935 | peptidyl-dipeptidase Dcp [EC:3.4.15.5] | 0,32+/-0,11 |
| K02823 | 0,025935 | dihydroorotate dehydrogenase electron transfer subunit | -0,14+/-0,05 |
| K06857 | 0,025959 | tungstate transport system ATP-binding protein [EC:7.3.2.6] | 1,32+/-0,47 |
| K03446 | 0,025977 | MFS transporter, DHA2 family, multidrug resistance protein | 0,72+/-0,26 |
| K01681 | 0,026048 | aconitate hydratase [EC:4.2.1.3] | -0,16+/-0,06 |
| K00568 | 0,026457 | 2-polyprenyl-6-hydroxyphenyl methylase / 3-demethylubiquinone-9 3-methyltransferase [EC:2.1.1.222 2.1.1.64] | 1,63+/-0,58 |
| K02474 | 0,026457 | UDP-N-acetyl-D-glucosamine/UDP-N-acetyl-D-galactosamine dehydrogenase [EC:1.1.1.136 1.1.1.-] | 0,54+/-0,19 |
| K06181 | 0,026466 | 23S rRNA pseudouridine2457 synthase [EC:5.4.99.20] | 1,35+/-0,48 |
| K00833 | 0,026466 | adenosylmethionine---8-amino-7-oxononanoate aminotransferase [EC:2.6.1.62] | 0,46+/-0,16 |
| K00817 | 0,026466 | histidinol-phosphate aminotransferase [EC:2.6.1.9] | -0,1+/-0,04 |
| K07053 | 0,026684 | 3',5'-nucleoside bisphosphate phosphatase [EC:3.1.3.97] | -0,15+/-0,05 |
| K01667 | 0,026709 | tryptophanase [EC:4.1.99.1] | 0,5+/-0,18 |
| K01082 | 0,026847 | 3'(2'), 5'-bisphosphate nucleotidase [EC:3.1.3.7] | 0,39+/-0,14 |
| K01712 | 0,026847 | urocanate hydratase [EC:4.2.1.49] | 0,39+/-0,14 |
| K00167 | 0,026847 | 2-oxoisovalerate dehydrogenase E1 component subunit beta [EC:1.2.4.4] | 1,55+/-0,55 |
| K06346 | 0,026847 | spoIIIJ-associated protein | -0,23+/-0,08 |
| K02556 | 0,027123 | chemotaxis protein MotA | -0,38+/-0,14 |
| K11717 | 0,027208 | cysteine desulfurase / selenocysteine lyase [EC:2.8.1.7 4.4.1.16] | 0,25+/-0,09 |
| K01468 | 0,027235 | imidazolonepropionase [EC:3.5.2.7] | 0,39+/-0,14 |
| K02040 | 0,027349 | phosphate transport system substrate-binding protein | -0,16+/-0,06 |
| K05341 | 0,027349 | amylosucrase [EC:2.4.1.4] | -0,41+/-0,15 |
| K00891 | 0,027351 | shikimate kinase [EC:2.7.1.71] | -0,16+/-0,06 |
| K03614 | 0,02741 | H+/Na+-translocating ferredoxin:NAD+ oxidoreductase subunit D [EC:7.1.1.11 7.2.1.2] | -0,18+/-0,07 |
| K00677 | 0,02741 | UDP-N-acetylglucosamine acyltransferase [EC:2.3.1.129] | 0,29+/-0,1 |
| K15554 | 0,027699 | sulfonate transport system permease protein | 0,84+/-0,3 |
| K03577 | 0,027699 | TetR/AcrR family transcriptional regulator, acrAB operon repressor | 1,93+/-0,7 |
| K02652 | 0,027699 | type IV pilus assembly protein PilB | 0,59+/-0,21 |
| K01876 | 0,028023 | aspartyl-tRNA synthetase [EC:6.1.1.12] | -0,11+/-0,04 |
| K09888 | 0,028038 | cell division protein ZapA | -0,15+/-0,05 |
| K03574 | 0,028156 | 8-oxo-dGTP diphosphatase [EC:3.6.1.55] | -0,21+/-0,08 |
| K10543 | 0,028297 | D-xylose transport system substrate-binding protein | 0,65+/-0,24 |
| K03832 | 0,02837 | periplasmic protein TonB | 0,28+/-0,1 |
| K06180 | 0,028467 | 23S rRNA pseudouridine1911/1915/1917 synthase [EC:5.4.99.23] | -0,13+/-0,05 |
| K06960 | 0,028675 | uncharacterized protein | -0,23+/-0,08 |
| K18672 | 0,028881 | diadenylate cyclase [EC:2.7.7.85] | -0,24+/-0,09 |
| K03771 | 0,028881 | peptidyl-prolyl cis-trans isomerase SurA [EC:5.2.1.8] | 0,32+/-0,12 |
| K01186 | 0,02895 | sialidase-1 [EC:3.2.1.18] | 0,46+/-0,17 |
| K01803 | 0,029253 | triosephosphate isomerase (TIM) [EC:5.3.1.1] | -0,1+/-0,04 |
| K03113 | 0,029489 | translation initiation factor 1 | 0,39+/-0,14 |
| K02655 | 0,029653 | type IV pilus assembly protein PilE | 1,1+/-0,4 |
| K03783 | 0,029876 | purine-nucleoside phosphorylase [EC:2.4.2.1] | -0,15+/-0,06 |
| K04750 | 0,029893 | PhnB protein | 0,87+/-0,32 |
| K07085 | 0,029893 | putative transport protein | 0,27+/-0,1 |
| K06195 | 0,030008 | ApaG protein | 1,72+/-0,63 |
| K05340 | 0,030008 | glucose uptake protein | 0,35+/-0,13 |
| K01752 | 0,030065 | L-serine dehydratase [EC:4.3.1.17] | -0,14+/-0,05 |
| K06919 | 0,03022 | putative DNA primase/helicase | -0,38+/-0,14 |
| K01580 | 0,030566 | glutamate decarboxylase [EC:4.1.1.15] | 0,51+/-0,19 |
| K15582 | 0,030661 | oligopeptide transport system permease protein | -0,27+/-0,1 |
| K09748 | 0,030903 | ribosome maturation factor RimP | -0,14+/-0,05 |
| K17319 | 0,031276 | putative aldouronate transport system permease protein | -0,37+/-0,14 |
| K07219 | 0,031276 | putative molybdopterin biosynthesis protein | 0,94+/-0,35 |
| K01089 | 0,031306 | imidazoleglycerol-phosphate dehydratase / histidinol-phosphatase [EC:4.2.1.19 3.1.3.15] | 0,32+/-0,12 |
| K01779 | 0,031423 | aspartate racemase [EC:5.1.1.13] | -0,34+/-0,13 |
| K04719 | 0,031508 | 5,6-dimethylbenzimidazole synthase [EC:1.13.11.79] | 2,12+/-0,78 |
| K12339 | 0,031576 | S-sulfo-L-cysteine synthase (O-acetyl-L-serine-dependent) [EC:2.5.1.144] | 0,71+/-0,26 |
| K00177 | 0,031576 | 2-oxoglutarate ferredoxin oxidoreductase subunit gamma [EC:1.2.7.3] | 0,35+/-0,13 |
| K00121 | 0,031576 | S-(hydroxymethyl)glutathione dehydrogenase / alcohol dehydrogenase [EC:1.1.1.284 1.1.1.1] | 1,08+/-0,39 |
| K01738 | 0,031576 | cysteine synthase [EC:2.5.1.47] | -0,14+/-0,05 |
| K06864 | 0,031605 | pyridinium-3,5-biscarboxylic acid mononucleotide sulfurtransferase [EC:4.4.1.37] | -0,26+/-0,09 |
| K19540 | 0,031919 | fructoselysine transporter | 1,41+/-0,52 |
| K00527 | 0,031919 | ribonucleoside-triphosphate reductase (thioredoxin) [EC:1.17.4.2] | -0,16+/-0,06 |
| K03570 | 0,031919 | rod shape-determining protein MreC | -0,14+/-0,05 |
| K01000 | 0,032054 | phospho-N-acetylmuramoyl-pentapeptide-transferase [EC:2.7.8.13] | -0,14+/-0,05 |
| K11896 | 0,032286 | type VI secretion system protein ImpG | 1,54+/-0,57 |
| K06023 | 0,032554 | HPr kinase/phosphorylase [EC:2.7.11.- 2.7.4.-] | -0,23+/-0,08 |
| K16937 | 0,032864 | thiosulfate dehydrogenase (quinone) large subunit [EC:1.8.5.2] | 0,66+/-0,25 |
| K15583 | 0,032948 | oligopeptide transport system ATP-binding protein | -0,23+/-0,09 |
| K01425 | 0,03301 | glutaminase [EC:3.5.1.2] | 0,5+/-0,18 |
| K09131 | 0,033076 | uncharacterized protein | 1,38+/-0,51 |
| K00171 | 0,033201 | pyruvate ferredoxin oxidoreductase delta subunit [EC:1.2.7.1] | 0,57+/-0,21 |
| K00700 | 0,033315 | 1,4-alpha-glucan branching enzyme [EC:2.4.1.18] | -0,16+/-0,06 |
| K03892 | 0,033423 | ArsR family transcriptional regulator, arsenate/arsenite/antimonite-responsive transcriptional repressor | -0,16+/-0,06 |
| K11895 | 0,033527 | type VI secretion system protein ImpH | 1,56+/-0,58 |
| K00874 | 0,033617 | 2-dehydro-3-deoxygluconokinase [EC:2.7.1.45] | -0,17+/-0,06 |
| K08191 | 0,033691 | MFS transporter, ACS family, aldohexuronate transporter | 0,33+/-0,12 |
| K03613 | 0,033783 | H+/Na+-translocating ferredoxin:NAD+ oxidoreductase subunit E | -0,17+/-0,06 |
| K03719 | 0,033957 | Lrp/AsnC family transcriptional regulator, leucine-responsive regulatory protein | -0,19+/-0,07 |
| K00937 | 0,033957 | polyphosphate kinase [EC:2.7.4.1] | -0,17+/-0,06 |
| K00363 | 0,034225 | nitrite reductase (NADH) small subunit [EC:1.7.1.15] | 1,23+/-0,46 |
| K09819 | 0,034506 | manganese/iron transport system permease protein | 1,57+/-0,58 |
| K04032 | 0,034538 | ethanolamine utilization cobalamin adenosyltransferase [EC:2.5.1.154] | 0,98+/-0,37 |
| K11103 | 0,034606 | aerobic C4-dicarboxylate transport protein | 1,25+/-0,46 |
| K03761 | 0,03482 | MFS transporter, MHS family, alpha-ketoglutarate permease | 1,7+/-0,64 |
| K00790 | 0,034876 | UDP-N-acetylglucosamine 1-carboxyvinyltransferase [EC:2.5.1.7] | -0,12+/-0,05 |
| K00248 | 0,035174 | butyryl-CoA dehydrogenase [EC:1.3.8.1] | -0,28+/-0,1 |
| K03060 | 0,03518 | DNA-directed RNA polymerase subunit omega [EC:2.7.7.6] | -0,2+/-0,07 |
| K01144 | 0,03518 | uncharacterized | 0,36+/-0,14 |
| K00940 | 0,03518 | nucleoside-diphosphate kinase [EC:2.7.4.6] | 0,29+/-0,11 |
| K10716 | 0,035395 | voltage-gated potassium channel | 0,52+/-0,19 |
| K02048 | 0,035395 | sulfate/thiosulfate transport system substrate-binding protein | -0,41+/-0,15 |
| K01091 | 0,035564 | phosphoglycolate phosphatase [EC:3.1.3.18] | -0,12+/-0,04 |
| K01938 | 0,035673 | formate--tetrahydrofolate ligase [EC:6.3.4.3] | -0,18+/-0,07 |
| K07467 | 0,035716 | putative DNA relaxase | -0,43+/-0,16 |
| K00847 | 0,035805 | fructokinase [EC:2.7.1.4] | -0,15+/-0,05 |
| K00942 | 0,036342 | guanylate kinase [EC:2.7.4.8] | -0,1+/-0,04 |
| K09794 | 0,036482 | uncharacterized protein | 1,22+/-0,46 |
| K01745 | 0,037261 | histidine ammonia-lyase [EC:4.3.1.3] | 0,38+/-0,14 |
| K01129 | 0,037261 | dGTPase [EC:3.1.5.1] | -0,18+/-0,07 |
| K02658 | 0,03768 | twitching motility two-component system response regulator PilH | 2,28+/-0,86 |
| K01840 | 0,03768 | phosphomannomutase [EC:5.4.2.8] | 0,31+/-0,12 |
| K15586 | 0,037752 | nickel transport system permease protein | 0,98+/-0,37 |
| K11934 | 0,037781 | outer membrane protein X | 0,67+/-0,25 |
| K01993 | 0,037781 | HlyD family secretion protein | 0,24+/-0,09 |
| K16055 | 0,037783 | trehalose 6-phosphate synthase/phosphatase [EC:2.4.1.15 3.1.3.12] | 0,61+/-0,23 |
| K02852 | 0,037885 | UDP-N-acetyl-D-mannosaminouronate:lipid I N-acetyl-D-mannosaminouronosyltransferase [EC:2.4.1.180] | 0,67+/-0,25 |
| K09924 | 0,037908 | uncharacterized protein | 0,61+/-0,23 |
| K10440 | 0,037974 | ribose transport system permease protein | -0,32+/-0,12 |
| K18702 | 0,038286 | CoA:oxalate CoA-transferase [EC:2.8.3.19] | 0,67+/-0,26 |
| K03154 | 0,038305 | sulfur carrier protein | -0,26+/-0,1 |
| K00818 | 0,038357 | acetylornithine aminotransferase [EC:2.6.1.11] | 0,29+/-0,11 |
| K01358 | 0,038509 | ATP-dependent Clp protease, protease subunit [EC:3.4.21.92] | -0,1+/-0,04 |
| K02275 | 0,038537 | cytochrome c oxidase subunit II [EC:7.1.1.9] | 1,12+/-0,42 |
| K01953 | 0,038981 | asparagine synthase (glutamine-hydrolysing) [EC:6.3.5.4] | -0,15+/-0,06 |
| K09811 | 0,039038 | cell division transport system permease protein | -0,14+/-0,06 |
| K02664 | 0,039467 | type IV pilus assembly protein PilO | 1,43+/-0,55 |
| K00782 | 0,039484 | L-lactate dehydrogenase complex protein LldG | 0,31+/-0,12 |
| K00666 | 0,039573 | fatty-acyl-CoA synthase [EC:6.2.1.-] | 0,48+/-0,18 |
| K09705 | 0,039742 | uncharacterized protein | 1,02+/-0,39 |
| K10907 | 0,039819 | aminotransferase [EC:2.6.1.-] | -0,22+/-0,08 |
| K02050 | 0,040265 | NitT/TauT family transport system permease protein | -0,13+/-0,05 |
| K10823 | 0,040277 | oligopeptide transport system ATP-binding protein | -0,22+/-0,09 |
| K09792 | 0,040931 | uncharacterized protein | 1,83+/-0,7 |
| K02377 | 0,04098 | GDP-L-fucose synthase [EC:1.1.1.271] | 0,4+/-0,15 |
| K01711 | 0,041321 | GDPmannose 4,6-dehydratase [EC:4.2.1.47] | 0,38+/-0,14 |
| K01719 | 0,041321 | uroporphyrinogen-III synthase [EC:4.2.1.75] | 0,33+/-0,13 |
| K16926 | 0,04137 | energy-coupling factor transport system substrate-specific component | -0,25+/-0,09 |
| K01429 | 0,041489 | urease subunit beta [EC:3.5.1.5] | 0,85+/-0,33 |
| K13990 | 0,041666 | glutamate formiminotransferase / formiminotetrahydrofolate cyclodeaminase [EC:2.1.2.5 4.3.1.4] | 0,54+/-0,21 |
| K10679 | 0,041704 | nitroreductase / dihydropteridine reductase [EC:1.-.-.- 1.5.1.34] | 0,78+/-0,3 |
| K10441 | 0,041714 | ribose transport system ATP-binding protein [EC:7.5.2.7] | -0,3+/-0,11 |
| K01940 | 0,04184 | argininosuccinate synthase [EC:6.3.4.5] | -0,12+/-0,05 |
| K13893 | 0,041917 | microcin C transport system substrate-binding protein | 1,73+/-0,67 |
| K01265 | 0,041941 | methionyl aminopeptidase [EC:3.4.11.18] | -0,11+/-0,04 |
| K00853 | 0,042422 | L-ribulokinase [EC:2.7.1.16] | 0,5+/-0,19 |
| K01058 | 0,042838 | phospholipase A1/A2 [EC:3.1.1.32 3.1.1.4] | 0,6+/-0,23 |
| K03119 | 0,042911 | taurine dioxygenase [EC:1.14.11.17] | 1,83+/-0,7 |
| K03980 | 0,042936 | putative peptidoglycan lipid II flippase | 0,43+/-0,17 |
| K19310 | 0,043073 | bacitracin transport system permease protein | -0,4+/-0,15 |
| K03168 | 0,043155 | DNA topoisomerase I [EC:5.6.2.1] | -0,12+/-0,05 |
| K02654 | 0,043231 | leader peptidase (prepilin peptidase) / N-methyltransferase [EC:3.4.23.43 2.1.1.-] | -0,23+/-0,09 |
| K03412 | 0,043231 | two-component system, chemotaxis family, protein-glutamate methylesterase/glutaminase [EC:3.1.1.61 3.5.1.44] | -0,37+/-0,14 |
| K03271 | 0,043426 | D-sedoheptulose 7-phosphate isomerase [EC:5.3.1.28] | 0,31+/-0,12 |
| K15587 | 0,044042 | nickel transport system ATP-binding protein [EC:7.2.2.11] | 0,96+/-0,37 |
| K01113 | 0,044403 | alkaline phosphatase D [EC:3.1.3.1] | 1,64+/-0,64 |
| K03338 | 0,044603 | 5-dehydro-2-deoxygluconokinase [EC:2.7.1.92] | 0,73+/-0,28 |
| K03436 | 0,044941 | DeoR family transcriptional regulator, fructose operon transcriptional repressor | -0,28+/-0,11 |
| K08602 | 0,045173 | oligoendopeptidase F [EC:3.4.24.-] | -0,38+/-0,15 |
| K10824 | 0,045474 | nickel transport system ATP-binding protein [EC:7.2.2.11] | 0,96+/-0,37 |
| K01610 | 0,045474 | phosphoenolpyruvate carboxykinase (ATP) [EC:4.1.1.49] | -0,16+/-0,06 |
| K03617 | 0,046152 | H+/Na+-translocating ferredoxin:NAD+ oxidoreductase subunit A | -0,14+/-0,06 |
| K18682 | 0,046176 | ribonucrease Y [EC:3.1.-.-] | -0,13+/-0,05 |
| K01810 | 0,04647 | glucose-6-phosphate isomerase [EC:5.3.1.9] | -0,09+/-0,04 |
| K00116 | 0,046736 | malate dehydrogenase (quinone) [EC:1.1.5.4] | 1,31+/-0,51 |
| K04518 | 0,046746 | prephenate dehydratase [EC:4.2.1.51] | 0,31+/-0,12 |
| K07775 | 0,047021 | two-component system, OmpR family, response regulator ResD | -0,34+/-0,13 |
| K07029 | 0,047031 | diacylglycerol kinase (ATP) [EC:2.7.1.107] | -0,3+/-0,12 |
| K03210 | 0,047038 | preprotein translocase subunit YajC | -0,1+/-0,04 |
| K01087 | 0,047258 | trehalose 6-phosphate phosphatase [EC:3.1.3.12] | 1,81+/-0,71 |
| K01197 | 0,047341 | hyaluronoglucosaminidase [EC:3.2.1.35] | 0,56+/-0,22 |
| K07263 | 0,047515 | zinc protease [EC:3.4.24.-] | 0,3+/-0,12 |
| K09954 | 0,047604 | uncharacterized protein | 1,72+/-0,68 |
| K08973 | 0,047784 | protoporphyrinogen IX oxidase [EC:1.3.99.-] | 1,93+/-0,76 |
| K00228 | 0,04786 | coproporphyrinogen III oxidase [EC:1.3.3.3] | 1,48+/-0,58 |
| K05995 | 0,04803 | dipeptidase E [EC:3.4.13.21] | -0,22+/-0,09 |
| K04091 | 0,048365 | alkanesulfonate monooxygenase [EC:1.14.14.5 1.14.14.34] | 1,64+/-0,65 |
| K00991 | 0,048402 | 2-C-methyl-D-erythritol 4-phosphate cytidylyltransferase [EC:2.7.7.60] | -0,14+/-0,05 |
| K01925 | 0,048422 | UDP-N-acetylmuramoylalanine--D-glutamate ligase [EC:6.3.2.9] | -0,1+/-0,04 |
| K18013 | 0,049278 | 3-keto-5-aminohexanoate cleavage enzyme [EC:2.3.1.247] | 0,55+/-0,22 |

**A**

**B**

**Figure S1. Associations between the CLR abundance of the four taxa and A) cytokine levels, and B) chemokine levels, in response to the preparation for the competition.** Pearson correlation was used for the analysis. Pre is before and Post after preparing for the competition. Change is the difference in values between Pre and Post. * P <.05
